# The effect of temperature, farm density and foot-and-mouth disease restrictions on the 2007 UK bluetongue outbreak

**DOI:** 10.1038/s41598-018-35941-z

**Published:** 2019-01-14

**Authors:** J. Turner, A. E. Jones, A. E. Heath, M. Wardeh, C. Caminade, G. Kluiters, R. G. Bowers, A. P. Morse, M. Baylis

**Affiliations:** 1 Department of Epidemiology and Population Health, Institute of Infection and Global Health, University of Liverpool, Leahurst Campus, Chester High Road, Neston, CH64 7TE UK; 2 Department of Epidemiology and Population Health, Institute of Infection and Global Health, University of Liverpool, Liverpool, L69 3GL UK; 3 School of Environmental Sciences, University of Liverpool, Liverpool, L69 7ZT UK; 4 NIHR, Health Protection Research Unit in Emerging and Zoonotic Infections, Liverpool, UK; 5 Department of Mathematical Sciences, University of Liverpool, Liverpool, L69 7ZL UK

## Abstract

**In 2006**, **bluetongue (BT)**, **a disease of ruminants**, **was introduced into northern Europe for the first time and more than two thousand farms across five countries were affected**. **In 2007**, **BT affected more than 35**,**000 farms in France and Germany alone**. **By contrast**, **the UK outbreak beginning in 2007 was relatively small**, **with only 135 farms in southeast England affected**. **We use a model to investigate the effects of three factors on the scale of BT outbreaks in the UK: (1) place of introduction; (2) temperature; and (3) animal movement restrictions**. **Our results suggest that the UK outbreak could have been much larger had the infection been introduced into the west of England either directly or as a result of the movement of infected animals from southeast England before the first case was detected**. **The fact that air temperatures in the UK in 2007 were marginally lower than average probably contributed to the UK outbreak being relatively small**. **Finally**, **our results indicate that BT movement restrictions are effective at controlling the spread of infection**. **However**, **foot-and-mouth disease restrictions in place before the detection and control of BT in 2007 almost certainly helped to limit BT spread prior to its detection**.

## Introduction

Bluetongue (BT), a vector-borne disease of ruminants (predominantly cattle and sheep), was first discovered in South Africa during the early 20^th^ century but has probably been endemic in Africa for hundreds if not thousands of years^1,2^. In recent years, climate change has enabled the Afrotropical vector, *Culicoides imicola* Kieffer (Diptera: Ceratopogonidae), to increase its range northwards^3^. It is now found in several southern European countries, where it has been implicated as the vector of numerous serotypes of BT virus (principally, BTV-1, BTV-2, BTV-4, BTV-9 and BTV-16). In addition, bluetongue virus (BTV) serotype 8 (BTV-8) was introduced to northern Europe in 2006 and native *Culicoides* species (four members of the Avaritia subgenus: *C*. *chiopterus*, *C*. *dewulfi*, *C*. *obsoletus*, *C*. *scoticus*; and two Pulicaris Group members: *C*. *pulicaris* and *C*. *punctatus*) were implicated as virus vectors^4,5^. A series of major BTV-8 outbreaks ensued, estimated to have cost over £100 million in the Netherlands alone^6^. These outbreaks included the first cases of BT reported in the UK, in 2007.

With high densities of competent native vectors throughout Europe and conditions right for the old-world tropical vector (*C*. *imicola*) in southern Europe, the probability of further outbreaks is high. BTV-8 re-emerged in France in 2015 after circulation had possibly been maintained in wild deer^7,8^. Control efforts have largely limited its spread to central France, though infected animals were detected on a farm in Normandy in May 2017, raising concerns in the UK about the possible incursion of infected vectors. Infected animals were imported into the UK from France in October 2017^9^. However, post-import testing identified these cases and no onward transmission was detected.

In preparation for future outbreaks in the UK and elsewhere, it is useful to study models of disease transmission and, in the case of vector-borne diseases, the vectors themselves. In previous work^10^, we developed a stochastic model of BTV transmission between farms in southeast England (the epicentre of the 2007 UK outbreak). Here we present results from an improved and extended model. In brief, we have (i) increased the area covered by the model so that it includes all farms with cattle and sheep in England and Wales; (ii) incorporated updated farm information, observed animal movement and daily temperature data; (iii) introduced a within-farm model that is used to simulate dynamic farm-specific prevalence, which in turn is used to estimate each farm’s individual degree of infectivity (i.e. which contributes to the risk it poses to other farms through the movement of animals); (iv) modelled between-farm vector transmission by considering the continuous release of infectious vectors from an infected farm and their diffusion and mortality. The model was validated using the 2007 UK outbreak data. The model itself and the significant improvements that have been made are described in further detail in the Electronic Supplementary Material (ESM).

One of the key drivers for improving the original model was to capture more accurately within-farm dynamics and vector dispersal, as local farm-to-farm transmission by vectors has been shown to account for most BT transmission^11–15^. However, it is important to understand the impact of animal movements too, as these can spread disease to other regions^13^. Once infection has been introduced to an area, factors such as temperature, farm density and number of animal movements influence the model outcome.

The UK outbreak of BT in 2007 was relatively small, with only 42 farms reporting disease at the time and an additional 93 detected by serology (25 through surveillance and tracing and the remainder as a result of pre-movement testing in 2008). By contrast, more than 2,000 farms were affected in Belgium, France, the Netherlands, Luxembourg and Germany in 2006, and more than 35,000 in France and Germany alone in 2007. It has not been explained why the UK outbreak was so much smaller than the ones in neighbouring countries, given that there are no known differences in vector species present in countries in northern Europe and they have similar farming systems.

Here we use the improved and extended model to investigate the effects of three factors on the scale of BT outbreaks in the UK: (1) the place of introduction, by varying the county into which the infection is introduced; (2) temperature, by using and comparing different years of temperature data; and (3) animal movement restrictions, by comparing three scenarios related to the UK outbreak in 2007, including one that incorporates the additional restrictions imposed after the discovery of foot-and-mouth disease virus, in Surrey, in August 2007.

## Results

### Place of introduction

**Figure 1:**
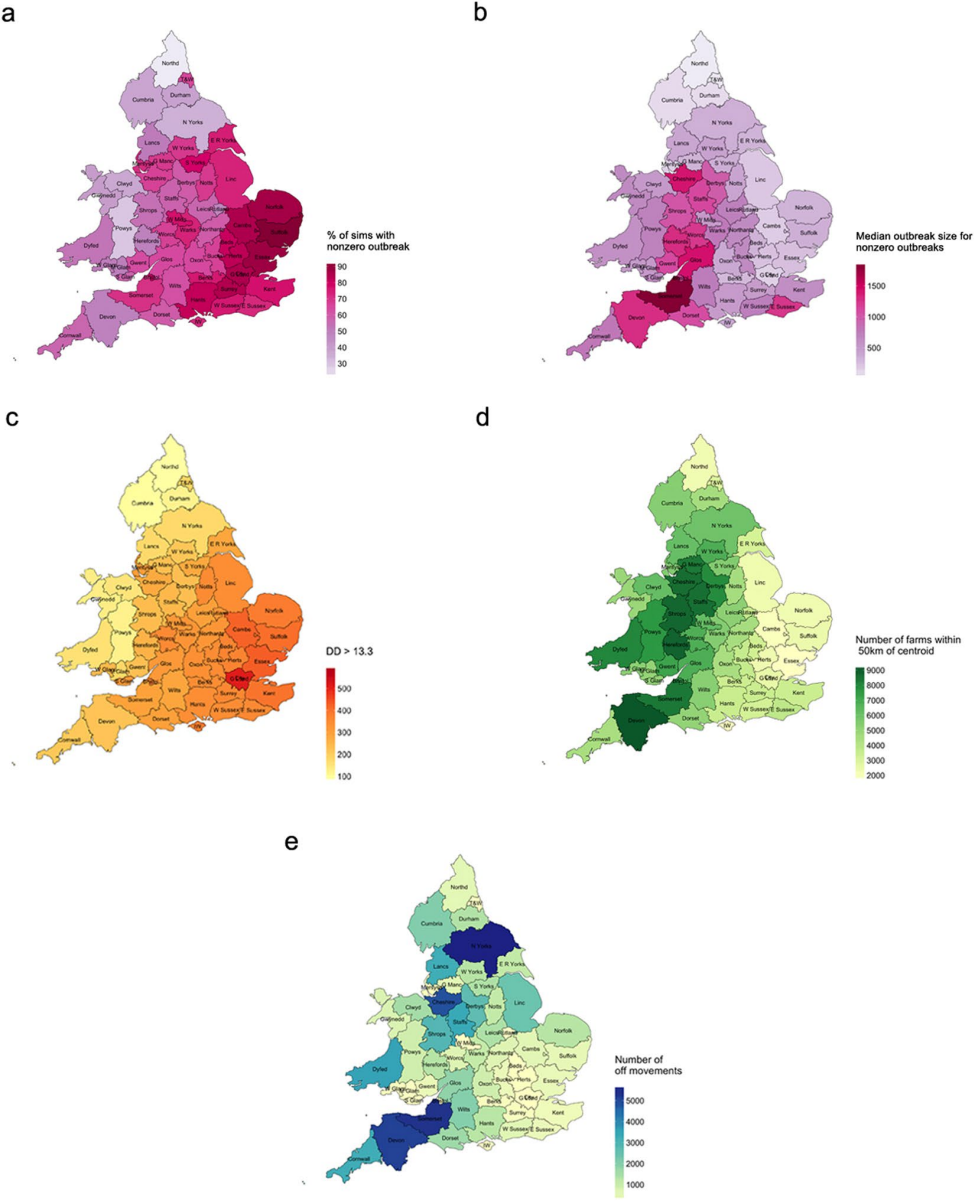
For each county: (**a**) percentage of simulations that result in an outbreak (i.e. at least one farm infected); (**b**) median outbreak size (median across non-zero values); (**c**) number of degree days above 13.3 °C; (**d**) number of farms within a 50 km radius of the centroid of the county polygon; (**e**) number of movements off farms in the county in June, July and August. Simulated results are based on 100 simulations per county. Maps were produced using RStudio v1.0.136 and R version 3.3.2 (www.r-project.org).

First we considered the effect of place of introduction of BTV on the final outbreak size. Figure 1a reveals the spatial distribution of the chance of an outbreak (i.e. the percentage of simulations which result in at least one farm becoming infected), while Fig. 1b shows the spatial distribution of the median outbreak size (in this case, the median across non-zero values only). The place of introduction was the county in which the index case was located, the index case being the infectious farm used to initiate a simulation. The results are based on 100 simulations per county. The plot reveals the variation in outbreak sizes between counties. The variation between simulations is shown in Figure S9.

Comparing Fig. 1a,c reveals that the chance of an outbreak is closely linked to the number of degree days^16^ above 13.3 °C (the minimum temperature required for virus development within the vector^17,18^ and hence the cut-off used within the model to allow/prevent potentially infectious vector activity). This is corroborated by the linear regression analysis of the percentage of simulations that result in an outbreak (labelled % sims, see Table 1). Model 1, which includes degree days above 13.3 °C as the only explanatory variable, explains nearly three quarters of the variance in % sims (R^2^ = 0.74). Comparing Fig. 1b,d suggests that median outbreak size is more closely related to farm density (here represented by the number of farms within 50 km of the centroid of the county polygon) than the number of degree days above 13.3 °C. Various combinations of degree days >13.3 °C, farm density and off-farm animal movements in June, July and August (JJA) were considered (Table 1). These explanatory variables were chosen because of their specific relevance to bluetongue virus transmission, but other related variables could be equally important. The results reveal that, for median outbreak size, farm density is the most important variable (R^2^ = 0.45). Adding degree days >13.3 °C improves the fit slightly (adjusted R^2^ = 0.56), while adding off-farm movements in JJA provides negligible additional improvement (adjusted R^2^ = 0.57). Overall, these results suggest that outbreaks are most likely to occur when infection is introduced into the East and South East of England, but that larger outbreaks could be expected, on average, if infection were introduced into the West Midlands and South West of England where farm density is typically higher. From Fig. 1c,d, it is clear that the number of DD >13.3 °C is negatively correlated with farm density (see also Table 2). Our results suggest that where sufficiently high values of each occur (e.g. in the counties of Cheshire, Herefordshire, Worcestershire, Gloucestershire and Somerset, where the number of DD >13.3 °C exceeds 275 and the number of farms within 50 km of the centroid of the county polygon exceeds 6800), the most favourable conditions for BT outbreaks are found.

**Table 1:** Results of linear regression analyses of measures of outbreak scale by county and explanatory variables relating to temperature, farm density and level of animal movements.

		Intercept	Coefficient of DD >13.3 °C	Coefficient of farm density	Coefficient of animal movements	R^2^	adj R^2^
		*β*_0_	*β*_1_	*β*_2_	*β*_3_		
**% sims**							
model 1	*y*_1_ = *β*_0_ + *β*_1_*x*_1_	19.58 (12.03, 27.13)	0.15 (0.13, 0.18)			0.74	0.74
model 2	*y*_1_ = *β*_0_ + *β*_1_*x*_1_ + *β*_2_*x*_2_	20.39 (8.00, 32.77)	0.15 (0.13, 0.18)	−0.0001 (−0.0013, 0.0011)		0.74	0.73
model 3	*y*_1_ = *β*_0_ + *β*_1_*x*_1_ + *β*_2_*x*_2_ + *β*_3_*x*_3_	20.36 (7.84, 32.89)	0.15 (0.12, 0.18)	−0.0001 (−0.0015, 0.0012)	0.00008 (−0.0018, 0.0020)	0.74	0.73
**median**							
model 1	*y*_2_ = *β*_0_ + *β*_1_*x*_1_	603.09 (226.29, 979.89)	0.04 (−1.20, 1.29)			0.00009	−0.019
model 2	*y*_2_ = *β*_0_ + *β*_1_*x*_1_ + *β*_2_*x*_2_	−723.47 (−1127.50, −319.44)	1.77 (0.85, 2.69)	0.16 (0.12, 0.20)		0.57	0.56
model 3	*y*_2_ = *β*_0_ + *β*_1_*x*_1_ + *β*_2_*x*_2_ + *β*_3_*x*_3_	−740.00 (−1137.69, −342.31)	1.90 (0.98, 2.81)	0.14 (0.10, 0.19)	0.05 (−0.01, 0.11)	0.60	0.57
model 4	*y*_2_ = *β*_0_ + *β*_2_*x*_2_	−40.49 (−256.76, 175.78)		0.13 (0.09, 0.17)		0.45	0.44
model 5	*y*_2_ = *β*_0_ + *β*_2_*x*_2_ + *β*_3_*x*_3_	−21.67 (−242.33, 198.99)		0.12 (0.07, 0.16)	0.03 (−0.04, 0.10)	0.46	0.44

The dependent variables are: *y*_1_, percentage of simulations with non-zero outbreak; *y*_2_, median of non-zero outbreak sizes. The independent explanatory variables are: *x*_1_, number of degree days >13.3 °C in the county; *x*_2_, number of farms within 50 km of county centroid; *x*_3_, total number of off-farm movements occurring in the county in June, July and August. 95% confidence intervals are given with each estimate.

**Table 2:** Partial rank correlation coefficients between characteristics of model input data (i.e. number of farms within 50 km of centroid, DD >13.3 °C and number of off-farm movements in Jun, Jul and Aug) and model outputs (i.e. percentage of simulations with nonzero outbreak and median of nonzero outbreak sizes).

Number of farms within 50 km of centroid	DD >13.3 °C	Number of OFF movements in Jun, Jul and Aug	Percentage of simulations with nonzero outbreak	median of nonzero outbreak sizes
1	−0.45	0.07	0.15	0.69
	1	−0.31	0.79	0.50
		1	0.11	0.23
			1	−0.30
				1

### Temperature

**Figure 2:**
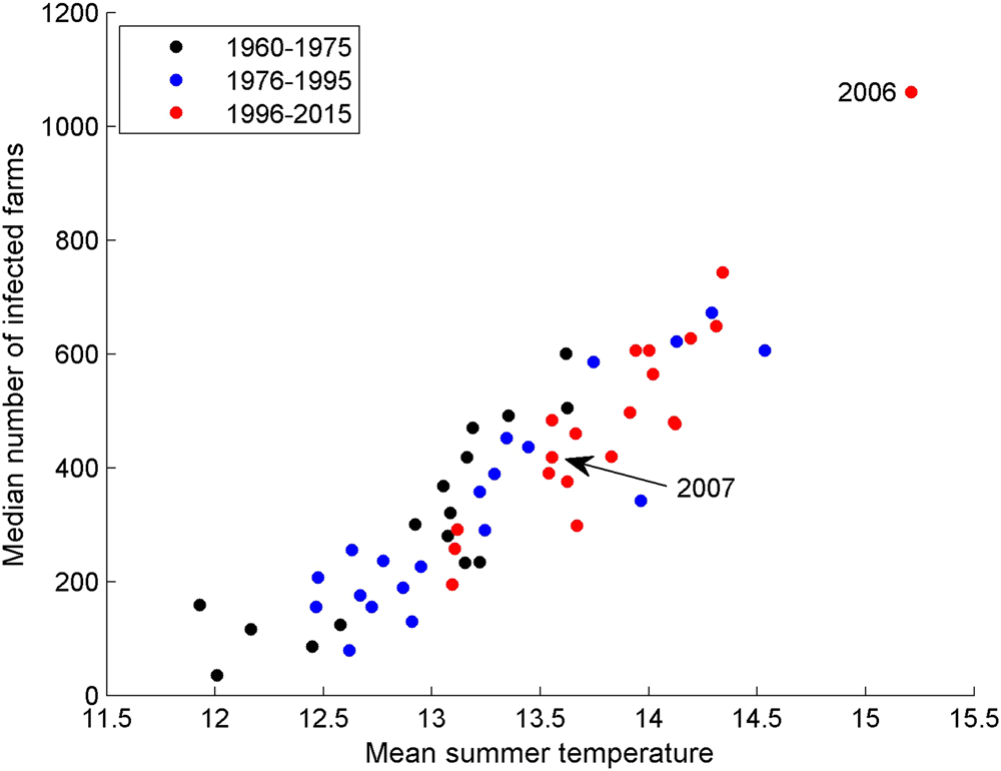
Relationship between mean summer temperature (°C) in England and Wales (1960 to 2015) and median number of farms infected (based on 100 simulations per year). Daily temperature data covering May to October for each grid point containing at least one farm with cattle and sheep were used to calculate the mean summer temperature in each case.

Figure 2 shows the strong relationship between simulated median outbreak size and summer temperature (correlation coefficient equals 0.91). From Fig. 2, it is clear that the summer of 2006 (the year of the first BTV outbreak in northern Europe) was exceptionally warm, while the summer of 2007 (the year of the UK BTV outbreak) was marginally cooler than the average over the last 10 years (Fig. S10a). The result of this in terms of simulated median outbreak size is a 2½-fold difference (i.e. 1061 to 420 infected farms). This suggests that the lower than average summer temperatures in 2007 could have been instrumental in limiting the size of the UK outbreak.

Importantly, outbreaks simulated using 2006 data are typically the largest of any year from 1960 to 2015 (Fig. S10b). Interestingly, as shown in Fig. 2, the simulated median outbreak size exceeds 500 farms seven times in the last 20 years (1996–2015), but only four times in the preceding 20 (1976–1995). This suggests that recent climate change would favour larger scale UK outbreaks.

### Animal movement restrictions

Finally, we turn our attention to animal movement restrictions, the main way of controlling an outbreak, particularly in the absence of a suitable vaccine, as in the UK in 2007.

**Figure 3:**
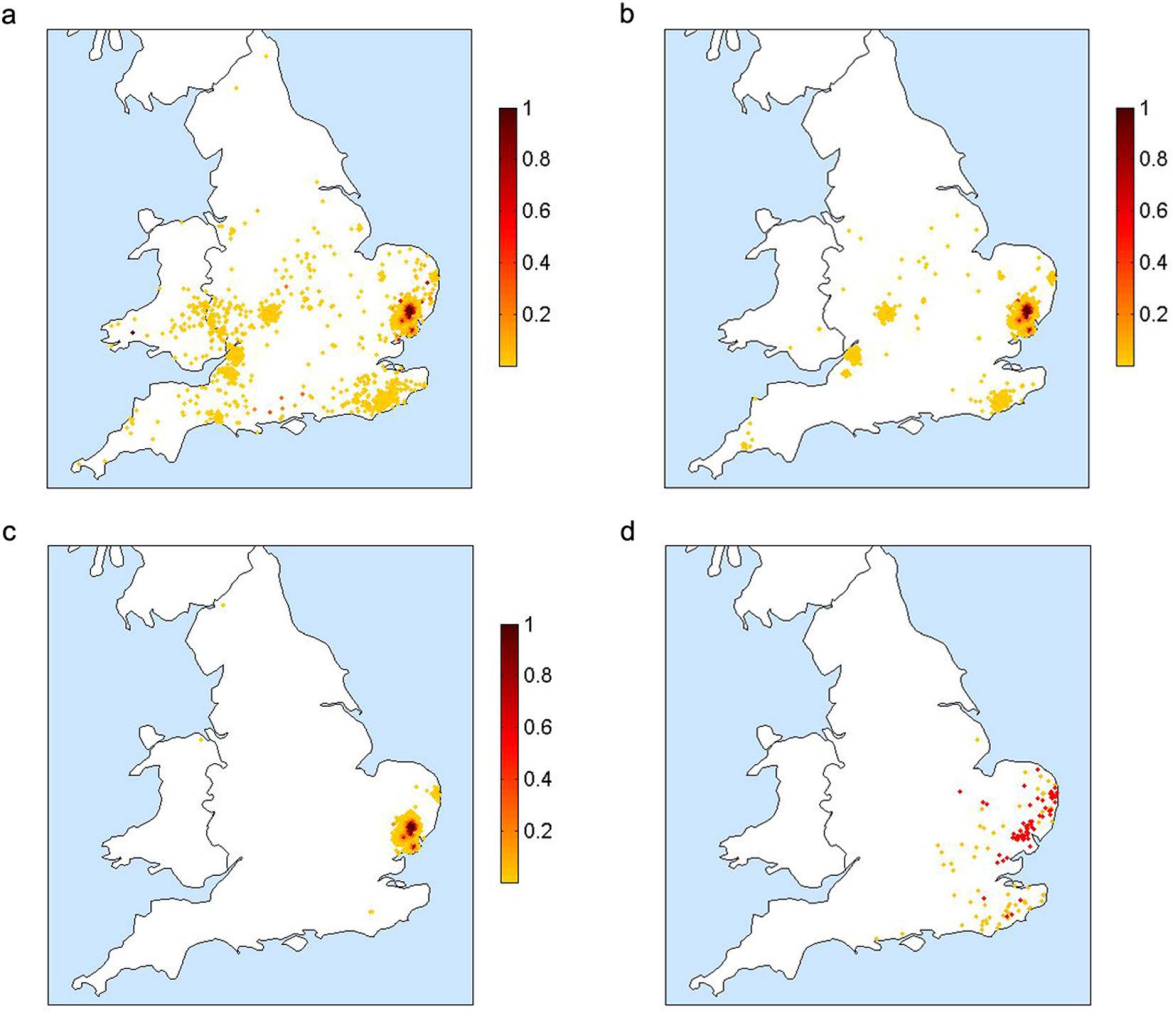
(**a**–**c**) Probability of infection (based on 500 simulations) for England and Wales when infection is introduced into Suffolk and Essex on 5 August: (**a**) No movement restrictions; (**b**) BTV movement restrictions only; (**c**) BTV and approximate FMD movement restrictions. (**d**) Spatial distribution of farms confirmed to be infected: red dots indicate farms with animals showing clinical signs or discovered as a result of surveillance on or before 31 December 2007; orange dots indicate farms discovered as a result of pre-movement testing between 1 January and 15 March 2008, but which are likely to have been infected during the previous vector season. Maps were produced using Matlab version 7.12.0.635 (R2011a, www.mathworks.com).

The results of the simulations are summarised as a ‘probability of infection’ (POI) plot, where each point represents a farm and the colour indicates the proportion of simulations in which that farm was infected. Figure 3a–c show the results for three different scenarios: (a) no movement restrictions; (b) BTV movement restrictions only; and (c) both BTV movement restrictions and approximate FMD movement restrictions (as described in ESM). Figure 3d shows the spatial distribution of farms infected during the UK 2007 outbreak. The red dots in Fig. 3d indicate farms that reported infection (i.e. animals showing clinical signs) or that were discovered as a result of surveillance on or before 31 December 2007, while the orange dots indicate farms that were discovered as a result of pre-movement testing (PMT) between 1 January and 15 March 2008. The latter have been included because it is likely that they were infected during the previous vector season. However, as midges have been found in barns during winter, transmission occurring between 1 January and 15 March 2008 cannot be completely excluded. Also, as animal movements were allowed within the restricted zone, it is possible that animals found to be seropositive by PMT were at a different location at the time they were infectious. The orange colouring is intended to reflect the uncertainty around these cases.

In all three simulated scenarios, there are examples of long-range transmission due to animal movements, which can either result in a single infected farm or a small cluster of infected farms if the local conditions are suitable for onward transmission. As the model is stochastic, the movement event that results in transmission is not the same in each simulation. This causes most farms to have a low POI compared to those close to the index cases which are infected in almost all simulations. In Fig. 3a, where spread is uncontrolled, we see many instances of long-range transmission with farms potentially anywhere in England and Wales being infected. Comparing Fig. 3a,b, we can clearly see that BTV movement restrictions are highly effective at reducing spread to other areas of the country. Without BTV movement restrictions, we found that the mean distance between an infected farm and the first index case could be as much as 180 km. With BTV movement restrictions, this figure reduced to 115 km, and it was just 15 km with both BTV and approximate FMD restrictions (Fig. 3c). The pattern in Fig. 3c is closer to the observed distribution of BT in 2007 (Fig. 3d) suggesting that FMD restrictions in place during the UK BTV outbreak could have contributed to containing the infection in the southeast of England. Without the additional (and earlier) restrictions for FMD, our model cannot readily account for the limited spatial distribution of BT in 2007.

## Discussion

Process-based simulation models such as the one presented here are recognised as valuable tools, enabling researchers to explore complex systems, consider different scenarios, investigate possible control measures and even estimate the additional impact of climate change on disease burden^19^. Our model has been developed for this purpose and used here to investigate the 2007 UK BTV outbreak.

Previous models of BT transmission in the UK have investigated different incursion scenarios^20,21^ and control strategies^22–24^ with incursion (and hence vaccination) generally limited to the south coast of England or the counties of Scotland bordering England^25^. Our results suggest that targeting vaccination in counties in the west of England, which were associated with the largest median outbreak sizes in our simulations, could reduce the potential for large outbreaks in England. This warrants further investigation as part of a cost-benefit analysis.

Other BT models have focussed on the role of vectors. Sumner *et al*.^13^ showed that 90% of BTV transmission between farms was due to vector dispersal and that the efficacy of animal movement restrictions depended on the way in which vector dispersal was modelled. Hendrickx *et al*.^11^ showed that short-range spread (<5 km) was driven by active vector flight, while Sedda *et al*.^12^ showed that 54% of new infections occurred within 5 km. Sedda *et al*.^12^ also showed that upwind and downwind vector movements contributed equally to disease spread. Our new model uses a diffusion-based process to replicate the random walk behaviour of vectors. The diffusion is two-dimensional, thereby allowing upwind and downwind vector dispersal. However, due to a lack of data for parameterisation, it does not take into account the influence of wind and hence cannot replicate any longer-range vector dispersal by this mechanism. In spite of this, the other features of the model (within-farm transmission model, more realistic vector to host ratio function that can be different for each farm, the ability to vaccinate individual farms, pre- and post-detection parameters, daily temperature data, etc.) mean that the current model is a great improvement on our earlier model and can be used to investigate a larger range of issues such as regional variations in transmission across the whole of England and Wales, targeted control strategies and the effects of future climate conditions as well as some of the reasons for the scale of the outbreak in the UK in 2007.

The UK outbreak in 2007 was small compared with the outbreak in northern Europe in 2006 and 2007. Our results suggest that several factors contributed to this, the first being the place of introduction. It is believed that BTV was introduced into southeast England on the night of 4^th^/5^th^ August 2007^26^. This area of England has a relatively low farm density. So, even though temperatures in this area were generally conducive to the development of infectious vectors (making an outbreak more likely), there were more limited opportunities for between-farm vector transmission, thereby restricting the size of the outbreak. Our results show that if the infection had been introduced into the west of England (either directly or as a result of animal movements from infected farms in southeast England before BTV was detected), then the outbreak would probably have been much larger, given the greater opportunities for between-farm vector transmission in this area and that the numbers of DD >13.3 °C in this area were not much lower than those in southeast England in 2007 (Fig. S11).

The second factor implicated in the relatively small size of the UK outbreak is temperature. It is generally believed that the unusually high temperatures seen in Europe in 2006 played a significant role in promoting transmission^27^. Our results for England and Wales indicate that the number of degree days above 13.3 °C (the virus’ development threshold in the midge vector) determines the probability that an outbreak spreads beyond the point of incursion and, to a lesser extent, the outbreak size. By driving the model with different years of temperature data, we were able to show a strong correlation between temperature and median simulated outbreak size and a trend towards larger outbreak sizes from 1960 to the present time, which follows the trend in increasing temperatures. The results also show that the marginally cooler than average summer temperatures seen in 2007 produce a median simulated outbreak size that is less than half the size expected with the summer temperatures observed in 2006. This clearly indicates that the UK outbreak would have been larger had the virus been introduced in a warmer year, something that is likely to occur more frequently in the future due to climate change.

The third factor is the presence of FMD movement restrictions. The effect of these in our model can be seen in Fig. 3. In the ESM, we discuss the possible reasons why the model predictions show a more clustered pattern of cases than that observed. These include a lack of illicit movements and movements from high risk to low risk zones, the effects of wind, the approximation of FMD restrictions and the omission of additional (i.e. on the same night) or subsequent introductions of infectious vectors. However, it is also worth remembering that Fig. 3a–c each summarise the results of 500 simulated outbreaks, whereas Fig. 3d depicts the result of a single real outbreak. In spite of this and the fact that there is some uncertainty in the exact distribution of farms infected with BTV during the 2007 UK outbreak, it is clear from Fig. 3 that the scenario with BTV and FMD restrictions produces a pattern that more closely matches the observed distribution. From this we can conclude that while BTV movement restrictions are effective at limiting long-range transmission of infection, the FMD restrictions that happened to be in place for seven weeks before BTV had been detected (and consequently before BTV movement restrictions could be implemented) almost certainly helped to contain the infection in the southeast of England. This is, to our knowledge, the first evidence suggesting that the UK’s FMD outbreak in 2007 impacted in any way on the subsequent BT outbreak.

To conclude, our results suggest that the relatively small size of the UK outbreak was partly due to when and where the infection was introduced and also to the fact that animal movement restrictions were already in place as a result of a different infection. It is easy to imagine that in future the UK may not be so lucky.

## Methods

### Model

The model was developed from an earlier stochastic simulation model described in Turner *et al*.^10^. In the model, farms (holdings with a unique CPH number) are divided into susceptible, exposed, infectious and detected states. Transmission between farms can occur as a result of the movement of exposed and infectious animals or the dispersal of infectious vectors. The first mechanism is modelled using recorded animal movements from a recent disease-free year (2006 or 2013, see *Data*). The second mechanism is driven by a diffusion-based function that is described in the ESM. Once a farm becomes infectious, the within-farm prevalence curve determines how infectious the farm is (on any given day after infection) to other farms through animal movement. Farm-specific prevalence curves are generated using a deterministic within-farm model (also described in the ESM). Outputs from the within-farm model also feed into the diffusion-based function controlling vector dispersal. Both the within-farm model and the main between-farm model include temperature-dependent functions, which in turn are driven by location-specific daily temperature data obtained from the UK Met Office. As with our earlier model, the main model includes animal movement restrictions based on those described in the GB Bluetongue Virus Disease Control Strategy. These have been updated to include the more stringent restriction, which prevents all movements within the inner (Control) zone. Our model also includes approximate FMD movement restrictions (as described in the ESM). Each of these restrictions can be applied independently. The default setting involves BTV movement restrictions only.

### Data

The model includes all farms containing cattle and sheep in England and Wales (approx. 100,000 farms). Cattle and sheep movement data and 2010 census data were obtained from the Animal and Plant Health Agency, APHA (formerly the Animal Health and Veterinary Laboratories Agency, AVHLA) Animal Movement Licensing System (AMLS) and Cattle Tracing System (CTS) databases. Animal movement data from 2006 (i.e. the closest disease-free year to the 2007 UK outbreak) were used for validation purposes and some simulations. The remaining simulations were produced using animal movements from a more recent disease-free year, namely 2013.

Historical daily temperature data for farm locations were extracted from the UKCP09 5 km daily observed archive^28^ for 1960 to 2015, by assigning farm temperature as the daily value of temperature at the grid point containing the farm. The UKCP09 dataset is derived from the UK Met Office archive of weather observations at synoptic stations, using regression to account for factors including altitude and coastal influence, and interpolation to transform the network values to a regular grid. A 5-day moving average (involving the current day and preceding four days) was applied to the farm-specific temperature data before use. As a result, values produced by the temperature-dependent functions of the model are less sensitive to daily variations in temperature.

### Validation

The model was validated in two stages. First, seroprevalence curves produced by the within-farm model for both BT and Schmallenberg (a different infection that affects the same hosts and is transmitted by the same vectors as BTV) were compared with within-farm data (see ESM for details). Secondly, the main between-farm model was validated using data from the 2007 UK BTV outbreak. For most of the model parameters, estimates could be obtained from the literature. However, there were three parameters, namely *p*_1_, *λ*_*D*0_ and *λ*_*D*1_, for which there was little or no information. These parameters were varied as part of the validation process and parameter sets that produced results consistent with the outbreak data were identified. A selection of parameter values was also considered during the sensitivity analysis. All details are given in the ESM.

### Sensitivity analysis

The sensitivity of the model to small perturbations in the parameter values was assessed by calculating the partial rank correlation coefficient (PRCC) of each parameter with respect to the median outbreak size. Additional simulations were used to investigate the specific effects of the number of bites per host per night and the ‘probability of detection’ too. The details and results are given in the ESM.

### Simulations

#### Place of introduction

To investigate regional differences in transmission, we varied the place of introduction of infection. We used Ordnance Survey Ceremonial Counties downloaded from OS OpenData. The place of introduction was defined as the county in which the index case was located, the index case being the infectious farm used to initiate a simulation. The results are based on 100 simulations per county. For each county, 100 farms were selected at random to be index cases and each used once. Infection was introduced on 1 June. Each simulation used the default parameter values shown in Table 3, BTV movement restrictions following detection of disease, animal movement data from 2013 (i.e. a recent disease-free year) and temperature data from 2011. Temperature data from 2011 was selected because the mean summer temperature (i.e. mean from May to October) in that year closely matched the median of summer values from 1996 to 2015. As such, 2011 can be considered to be an average year.

**Table 3:** BTV model functions, parameters and default values. Most were obtained from the literature (Gubbins *et al*.^18^ and references therein). Others were estimated during validation (see ESM for details). For a full description of the model see ESM and^10^.

Description	Parameters	Default values
Vector to host ratio *m*(*t*,*T*) = exp(*b*_0_ + *p*_1_sin(*θ*(*t − ψ*_1_)) + *p*_2_sin(2*θ*(*t − ψ*_2_)) + *cT*), where *t* = time *T* = temperature	*b*_0_, *p*_1_, *p*_2_, *c*, *θ*, *ψ*_1_, *ψ*_2_	0, 10.59, 3.71, 0.07, 0.0172, 128.4, 81.7
Extrinsic incubation rate *ν*(*T*) = max(0, *ν*_1_(*T* − *ν*_2_))	*ν*_1_, *ν*_2_	0.019, 13.34
Biting rate *a*(*T*) = max (0, *a*_1_*T*(*T* − *a*_3_) (*a*_4_ − *T*))^1/*a*2^	*a*_1_, *a*_2_, *a*_3_, *a*_4_	0.0002, 2.7, 3.7, 41.9
Probability of transmission from vector to host	*β*_*vh*_	0.9
Vector mortality rate *μ*(*T*) = *μ*_1_ exp (*μ*_2_*T*)	*μ*_1_, *μ*_2_	0.009, 0.16
Host incubation rate where subscripts are *C* for cattle *S* for sheep	*c*_*C*_, *c*_*S*_	1/7, 1/5
Probability of transmission from host to vector	*β*_*hv*_	0.01
Host recovery rate where subscripts are *C* for cattle *S* for sheep	*r*_*C*_, *r*_*S*_	1/20.6, 1/16.4
Vector activity threshold (equals *ν*_2_)	*T*_*cutoff*_	13.34
Diffusion parameter	*D*	0.531
Feeding preference	*σ*	0.5
Probability of conversion $1-\exp \,(\,-\,{({T}_{\exp }/{\lambda }_{C})}^{{k}_{C}})$ where *T*_exp_ = time since farm exposed	*λ*_*C*_, *k*_*C*_	10, 3
Proportion exposed (still needed for *E* farms; for *I* farms, prevE taken from ODE model)	prevE	0.01
Probability of detection 1 − (1 − *λ*_*A*_*d*_*I*_)^*H*^, where *λ*_*A*_ = *λ*_*D0*_ (or *λ*_*D1*_) for cattle and mixed farms prior to (or after) detection of the first case. Similarly for sheep with *λ*_*DS0*_ and *λ*_*DS1*_. *d*_*I*_ = prevalence of infection on the farm *H* = herd size	*λ*_*D0*_, *λ*_*DS0*_, *λ*_*D1*_, *λ*_*DS1*_	0.001, 0.001, 0.01, 0.01
Degree of susceptibility $1-(1-({d}_{S0}-(F/H)))\,\exp \,(\,-\,{({T}_{rec}/{\lambda }_{I})}^{{k}_{I}})$ where *d*_*S*0_ = farm’s degree of susceptibility prior to its last outbreak of infection *F* = number of animals on farm infected in last outbreak *T*_*rec*_ = time since farm recovered	*λ*_*I*_, *k*_*I*_	912.5, 2
Movement restriction zones (radius in km)	CZrad1, PZrad1, SZrad1	20, 100, 150
Maximum distance a vector can fly unassisted in a day (km)	vecd1	15
Proportion of cattle in a mixed herd	mixed_ratio	0.33

#### Temperature.

The effect of temperature was investigated using data from individual years from 1960 to 2015. To minimise the effect of place of introduction, each simulation (one hundred per year) was initiated using a single randomly-selected index case in Hampshire, southern England. As before, infection was introduced on 1 June and each simulation used the default parameter values shown in Table 3, BTV movement restrictions following detection of disease and animal movement data from 2013.

#### Animal movement restrictions.

In order to focus on the situation in the UK in 2007, we used temperature data from 2007 and initiated each simulation with nine latently-infected index cases in Suffolk and Essex (the number of farms estimated to have been infected by infectious midges from continental Europe; see further details in the ESM). The time of introduction was 5 August, the likely date of introduction into the UK, as suggested by Gloster *et al*.^26^. Each simulation (500 per POI plot) used the default parameter values shown in Table 3 and animal movement data from 2006 (i.e. the closest disease-free year to the 2007 UK BTV outbreak). Three sets of movement restrictions were considered, namely (i) no restrictions, (ii) BTV movement restrictions only and (iii) BTV and approximate FMD movement restrictions.

### Code availability

The full model code in C++ is available upon request by contacting JT, AEJ or MB.

## Electronic supplementary material

Electronic Supplementary Material

## Data Availability

Temperature data are only available from the UK Met Office. The UKCP09 daily gridded climate dataset from 1960 to 2011 is publicly available, following registration, at [https://www.metoffice.gov.uk/climatechange/science/monitoring/ukcp09/download/index.html]. The UKCP09 daily gridded climate dataset from 2012 to 2015 is available upon reasonable request from the National Climate Centre at the UK Met Office and subject to a confidentiality agreement. Data on farm locations, animal movements and BTV cases are only available from the APHA and subject to a confidentiality agreement. Ordnance Survey boundary data is publicly available at [https://www.ordnancesurvey.co.uk/opendatadownload/products.html#BDLINE]. Model output (other than that which could be used to identify individual farms) are available from the University of Liverpool repository at [10.17638/datacat.liverpool.ac.uk/620].
