# Supplementary material for: The effect of temperature, farm density and foot-and-mouth disease restrictions on the 2007 UK bluetongue outbreak
Source: Sci Rep. 2019 Jan 14;9:112. doi: 10.1038/s41598-018-35941-z (PMC6331605; doi:10.1038/s41598-018-35941-z)
Supplement: Supplementary file 1 — Electronic Supplementary Material [file 41598_2018_35941_MOESM1_ESM.pdf]

Electronic supplementary material

The effect of temperature, farm density and foot-and-mouth disease restrictions on the 2007 UK bluetongue outbreak

J. Turner, A.E. Jones, A. Heath, M. Wardeh, C. Caminade, G. Kluiters, R.G. Bowers, A. Morse, M. Baylis.

S1. Model developments

The model was developed from an earlier stochastic model [S1] and incorporates several significant improvements. In the model, farms (holdings with a unique CPH number) are divided into susceptible, exposed, infectious and detected states. Transmission between farms can occur as a result of the movement of exposed and infectious animals or the dispersal of infectious vectors. The first mechanism is modelled using recorded animal movements from a recent disease-free year (2006 or 2013, details below). The second mechanism uses a diffusion-based function that is described in section S1.4. Once a farm becomes infectious, the within-farm prevalence curve determines how infectious the farm is (on any given day after infection) to other farms through animal movement. Farm-specific prevalence curves are generated using a deterministic within-farm model, as described in section S1.2.2. Outputs from the within-farm model also feed into the diffusion-based function controlling vector dispersal. Both the within-farm model and the main between-farm model include temperature-dependent functions, which in turn are driven by location-specific daily temperature data. As with our earlier model, the main model includes animal movement restrictions based on those described in the GB Bluetongue Virus Disease Control Strategy. These have been updated to include the more stringent restriction, which prevents all movements within the inner (Control) zone. Our model also includes approximate FMD movement restrictions (as described in S2.2). Each of these restrictions can be applied independently. The default setting involves BTV movement restrictions only. Full details of all improvements are given below.

S1.1 Data extended and updated

The model is coded in C++ and includes all farms containing cattle and sheep in England and Wales (approx. 100,000 farms). Cattle and sheep movement data and 2010 census data were obtained from the Animal and Plant Health Agency (formerly the Animal Health and Veterinary Laboratories Agency, AVHLA) Animal Movement Licensing System (AMLS) and Cattle Tracing System (CTS) databases. The data were subsequently cleaned and stored in an SQL Server 2008 database.

S1.1.1 Farms

Farm data were collated from four sources: 2010 census data, cattle population data collated for each farm during the month of June (2001 – 2014), location data associated with cattle movement datasets and sheep movement datasets. CPH (county parish holding) number was used as a unique identifier to link the farms across the various sources. Location data were consolidated from the four sources so they were as accurate as possible.

Due to missing data, calculation of herd sizes for each farm were separated into multiple levels:

1. Census data: where available, 2010 census data was used as primary source for herd sizes for both sheep and cattle.
2. Population data associated with cattle movement: provided source for cattle population of the farms.

For each farm, the following data items were extracted:

1. ID: unique identifier of the farm
2. CPH: county parish holding (CPH) number of the farm.
3. X: x coordinate of the farm.

4. Y: y coordinate of the farm.
5. Postcode: post code associated with the farm.
6. County: county of the farm.
7. Country: country of the farm (England, Wales, Northern Ireland or Scotland). Only Welsh and English farms were included in the model.
8. Address: address of the farm.
9. CTS: numeric variable indicating whether the farm has cattle movement associated with it in the given year of analysis. -1 = no movement was recorded, 0 = movement recorded in different year, 1=movement recorded in the given year.
10. AMLS: numeric variable indicating whether the farm has sheep movement associated with it in the given year of analysis. -1 = no movement was recorded, 0 = movement recorded in different year, 1=movement recorded in the given year.
11. CensusCattle: number of cattle on the farm as identified in the 2010 census.
12. CensusSheep: number of sheep on the farm as identified in the 2010 census.
13. HS_01 : size of the farm's herd in 2001, if known (cattle only).
14. HS_02 : size of the farm's herd in 2002, if known (cattle only).
15. HS_03 : size of the farm's herd in 2003, if known (cattle only).
16. HS_04 : size of the farm's herd in 2004, if known (cattle only).
17. HS_05 : size of the farm's herd in 2005, if known (cattle only).
18. HS_06 : size of the farm's herd in 2006, if known (cattle only).
19. HS_07 : size of the farm's herd in 2007, if known (cattle only).
20. HS_08 : size of the farm's herd in 2008, if known (cattle only).
21. HS_09 : size of the farm's herd in 2009, if known (cattle only).
22. HS_10 : size of the farm's herd in 2010, if known (cattle only).
23. HS_11 : size of the farm's herd in 2011, if known (cattle only).
24. HS_12 : size of the farm's herd in 2012, if known (cattle only).
25. HS_13 : size of the farm's herd in 2013, if known (cattle only).
26. HS_14 : size of the farm's herd in 2014, if known (cattle only).
27. Ignore: logical variable indicating if the farm should be left out of the analysis due to insufficient data (e.g. missing location data, missing movement data), or being in the wrong location (e.g. in the sea, in Scotland).

S1.1.2 Direct movements

Direct movements were extracted for each year of the analysis for both cattle and sheep from the underlying database as follows:

1. Cattle: individual cattle movements between farms (i.e. the departure and destination locations were both farms) were aggregated from the underlying database for each pair of farms for each day of the analysis year.
2. Sheep: movements of sheep between farms, whereby the departure and destination locations were both farms, were extracted from the database for each pair of farms for each day of the analysis year.
3. The two datasets were then merged, so the total number of animals moved between each two farms for each day equalled the number of both cattle and sheep moved.

S1.1.3 Indirect movements

Indirect movements were extracted for sheep from the underlying database as follows:

1. Total number of sheep moved from farms to markets was aggregated from the database for each farm and market for day of the analysis year.
2. Total number of sheep moved from markets to farms was aggregated from the database for each market and farm for day of the analysis year.

The mixing mechanism that assigns farm to farm links when sheep are moved via markets has not changed and a value of q = 0.5 was used for this paper.

S1.1.4 Temperatures

Historical daily temperature data for farm locations were extracted from the UKCP09 5km daily observed archive [S2] for 1960 to 2015, by assigning farm temperature as the daily value of temperature at the grid point containing the farm. The UKCP09 dataset is derived from the UK Met Office archive of weather observations at synoptic stations, using regression to account for factors including altitude and coastal influence, and interpolation to transform the network values to a regular grid. Note that a 5-day moving average (involving the current day and preceding four days) was applied to the farm-specific temperature data before use. As a result, values produced by the temperature-dependent functions of the model are less sensitive to daily variations in temperature.

S1.2 Farm-specific values introduced

S1.2.1 Degree of susceptibility

Degree of susceptibility (d_S) values are an important factor in the calculation of transmission probabilities. In the previous model, all farms were assumed to be fully-susceptible at the start. The value for a farm would be updated after infection and, as the total number of infected animals (final size) on the farm was unknown, the maximum prevalence achieved on the farm was used. With the introduction of the within-farm model, it is now possible to calculate the final size of the outbreak on each farm separately and adjust each farm's d_S value accordingly, thereby correctly estimating each farm's individual re-infection risk. Also, in the new model, each farm is given a d_S value at the start. These can all be 100% (i.e. fully susceptible), as in the previous model. Alternatively, they can take different values that represent the proportion of animals on each farm that are protected from infection by either acquired immunity or vaccination.

S1.2.2 Prevalence curve

Farm-specific prevalence curves were introduced using a within-farm model. Although the main model is stochastic, the within-farm model was chosen to be deterministic for computational reasons. The model is a two-host, one-vector model with host incubation and recovery (i.e. SEIR - type) and vector incubation (i.e. SEI - type). The host population is assumed to be constant, while the vector population varies seasonally (driven by a temperature-dependent recruitment rate that is related to the new vector to host ratio function described in section S1.3). The model equations are given below.

Hosts ($i = C$ for cattle or S for sheep)

Number susceptible $$\frac{dX_i}{dt} = -\lambda_i(T,t)X_i$$

Number exposed $$\frac{dE_i}{dt} = \lambda_i(T,t)X_i - c_iE_i$$

Number infected $$\frac{dY_i}{dt} = c_iE_i - r_iY_i$$

Number recovered $$\frac{dZ_i}{dt} = r_iY_i$$

Total number $$\frac{dH_i}{dt} = 0$$

Force of infection $$\lambda_i(T,t) = \beta_{vh}a(T)\phi_i m_i(t)\frac{I}{N}$$

where $a(T) = \max(0, 0.0002T(T-3.7)(41.9-T)^{1/2.7})$,

$$\phi_C = \frac{H_C}{H_C + \sigma H_S} = \phi \quad \text{and} \quad \phi_S = 1 - \phi,$$

$$m_C(t) = N / H_C \quad \text{and} \quad m_S(t) = N / H_S,$$

$$m(t) = \frac{N}{H} = \exp\left(b_0 + p_1 \sin(\theta(t - \psi_1)) + p_2 \sin(2\theta(t - \psi_2)) + cT\right).$$

Vectors:

Number susceptible

$$\frac{dS}{dt} = (\theta p_1 \cos(\theta(t - \psi_1)) + 2\theta p_2 \cos(2\theta(t - \psi_2)) + c(T_{end} - T_{start}) + \mu(T))N...$$

$$...- \lambda_V(T,t)S - \mu(T)S$$

Number latent $\frac{dL}{dt} = \lambda_V(T,t)S - (\nu(T) + \mu(T))L$

Number infectious $\frac{dI}{dt} = \nu(T)L - \mu(T)I$

Total number $\frac{dN}{dt} = N\left(\theta p_1 \cos(\theta(t - \psi_1)) + 2\theta p_2 \cos(2\theta(t - \psi_2)) + c(T_{end} - T_{start})\right)$

Force of infection $\lambda_V(T,t) = \beta_{hv} a(T)\left[\phi \frac{Y_C}{H_C} + (1 - \phi)\frac{Y_S}{H_S}\right]$

where $\mu(T) = 0.009\exp(0.16T)$,

$\nu(T) = \max(0, 0.019(T - 13.34))$.

The parameters ν, a and μ are temperature-dependent functions. The vector to host ratio *m* is both time- and temperature-dependent. Temperature estimates (derived from real data) for the start and end of each daily time step are read in at the start. Temperature then varies linearly between T_{start} and T_{end} for that day. The previous recruitment rate ρ is replaced with $\theta p_1 \cos(\theta(t - \psi_1)) + 2\theta p_2 \cos(2\theta(t - \psi_2)) + c(T_{end} - T_{start}) + \mu(T)$, leading to the new *m* function used in main code.

The within-farm model is used to produce a prevalence curve for each farm that reflects the number of animals on that farm, the location (and hence temperature profile) for that farm and the time of year that the infection was introduced to that farm. Using the number of days since infection was introduced, we can obtain from the prevalence curve a measure of the farm's infectivity (d_l), which indicates how infectious the farm is to other farms. Figure S1 shows the range of prevalence curves that could occur during a single simulation when infection is introduced on 15th of each month from May to October. The curve labelled 'mean all' is the mean across years (1990 – 2015) when each year consists of a time series created by taking the mean across all farm grid points. The curve labelled '2006 all' shows the mean across all farm grid points for year 2006 only (i.e. the year of the first BTV outbreak in northern Europe). The other curves are described in the figure legend.

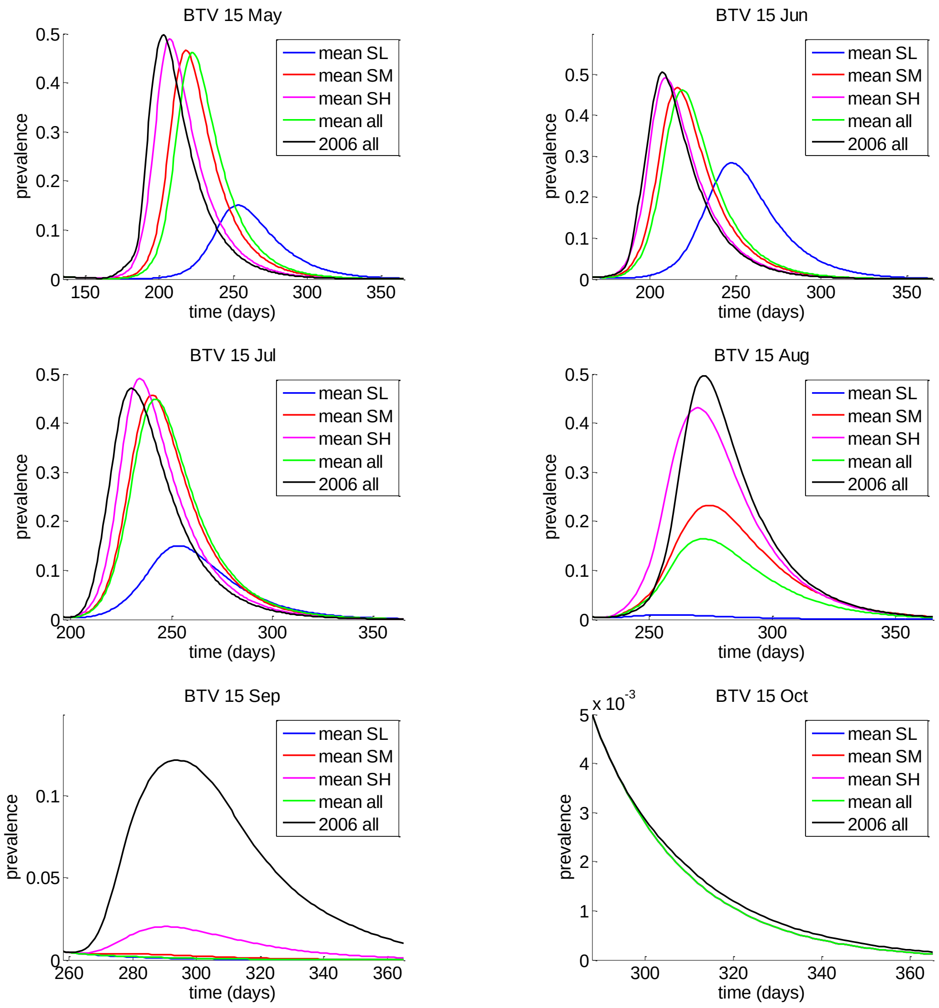

Figure S1: Range of prevalence curves that could occur during a single simulation with *ma* ≈ 500. Date of introduction is shown in the title of each subfigure. Curves were generated using temperature data from 1990 – 2015. Curve 'mean all' is the mean across years when using the mean across all farm grid points. Curve 'mean SL' is the mean across years when using a randomly-chosen farm grid point whose Jun-Jul-Aug mean temperature falls within the low (0-0.2) quantile band. Curves 'mean SM' and 'mean SH' are the equivalent curves for the middle (0.4-0.6) and high (0.8-1) quantile bands, respectively. Curve '2006 all' shows the mean across all farm grid points for year 2006 only.

As the within-farm model is only used to produce a prevalence curve for a farm once that farm has become infectious, and our definition of infectious is having at least one infectious animal, the within-farm model is always initiated with one infectious animal (a cow if the farm is classed as cattle or mixed and a sheep otherwise). Using one exposed animal or one infectious vector instead produces an almost identical curve. The curves in Figure S1 were produced using the default parameter values shown in Table 3 in the main manuscript. These produce summer *ma* (i.e. the product of *m* and *a*) values that are approximately equal to 500. As the model is very sensitive to the size of *ma*, alternative values were considered when analysing the main (i.e. between-farm) model (see S3.1).

The within-farm model also provides farm-specific time series giving the number of infectious vectors released each day. These are used to calculate the risk to nearby farms resulting from vector dispersal, as described below in section S1.4.

The movement of exposed (i.e. latently-infected) animals is also a route of transmission. As in the previous model, the proportion of exposed animals on an exposed farm is assumed to be 0.01. For infected and detected farms, this was previously increased to 0.02. However, the proportion of exposed animals on these farms is now drawn from the within-farm model (by generating a 'proportion exposed' curve at the same time as the prevalence curve).

S1.2.3 Vector to host ratio

The model has been modified so that different parameter values for the vector to host ratio (*m*) function can be specified for each farm.

S1.3 Vector to host ratio function replaced

A new seasonal vector-to-host ratio (*m*) function has been introduced. It is a simplified version of that presented in Sanders et al. [S3]. It produces a seasonal pattern of *m* values that, when combined with the temperature-dependent biting rate (*a*), reasonably represents the pattern of trap catch data [S3,S4], in particular the summer plateau so often observed. Note that we have assumed that a trap acts like a host, attracting vectors that are looking to feed. Therefore, the number of vectors caught each night represents the number of vectors per host per night that are seeking to feed. We take this to represent the number of bites per host per night (i.e. number of vectors per host (*m*) x number of bites per vector per night (*a*)).

The model is highly sensitive to *m*, and hence *ma*, so a range of parameter values were considered, corresponding to *ma* plateau values equal to 100, 500 and 1000. The results are shown below in section S3.1.

S1.4 Distance kernel replaced

The distance kernel that controls between-farm vector transmission has been replaced with one determined by continuous release, diffusion and mortality of infectious vectors. The details are given below along with the new 'probability of exposure by vector movement' calculation, which had to be modified as a result of the changes.

The basis for our calculation is a standard result for diffusion in two dimensions with additional decay [S5]. The concentration at distance *r* and time *t*, *C*(*r*,*t*), from a point source on an infinite plane surface is given by

$$C(r,t)=\left(\frac{M}{4\pi Dt}\right)\exp\left(\frac{-r^2}{4Dt}-\mu t\right),$$

where *M* is the total number added at the start, *μ* is the per capita mortality rate of infectious vectors and *D* is the diffusion coefficient (see equation 1 below). If the source is continuous, rather than instantaneous, specifically if vectors emanate at rate *ω*(*t*), then the concentration is found by integrating the instantaneous solution with respect to time *t* [S5 p. 31 – 32].

$$C(r,t)=\int_0^t \frac{\omega(t')}{4\pi D(t-t')}\exp\left(\frac{-r^2}{4D(t-t')}-\mu(t-t')\right)dt'.$$

Unfortunately, in this case an algebraic solution is not possible, not least because *μ* changes daily with the discrete temperature data. However, we can calculate a discrete approximation by treating each day as a separate instantaneous release and then summing across the different days. The sum can be written as

$$C(r,t)=\sum_{i=0}^{t-1}C_i(r,t),$$

where

$$C_i(r,t)=\frac{\omega(i)}{4\pi D(t-i)}\exp\left(\frac{-r^2}{4D(t-i)}-\sum_{j=3}^{t-1}\mu_j\right).$$

Note that in the discrete case, we stop at t - 1 (i.e. the last day that vectors are released before time t).

The accuracy of the approximation can be increased by dividing each day into a number of sub-steps (nsteps). The higher the number of sub-steps, the better the approximation. Unfortunately, increasing the number of sub-steps also increases computation time. As shown in Figure S2, 10 sub-steps was found to be sufficient for a good approximation, with accuracy only an issue for small distances (e.g. < 0.5km).

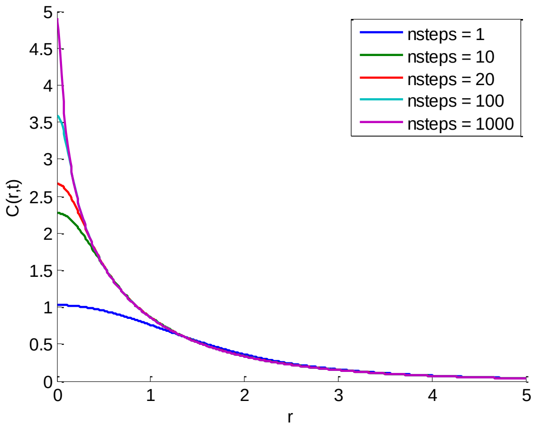

Figure S2: $C(r,t)$ plotted against r. The exact solution tends to infinity as r tends to zero.

Within the main code, the $C(r,t)$ function is evaluated for different T_{inf} (i.e. time since the source farm became infectious) and different r (i.e. distance from source to target farm). The values for $\omega(t')$ (i.e. the number of new infectious vectors released on day t') are drawn from the within-farm model, as mentioned earlier. Each mortality rate μ_i is the average mortality rate for time step i to i + 1. The advantage of this approach over the previous distance kernel approach that involved the relatively arbitrary parameter α_2 is that the remaining parameter D can be estimated as follows from data on the distance travelled by a vector in t days after its release. Let x_t be a vector containing the distances from the source farm that each vector travelled in time t (assuming random walk motion). For diffusion in two-dimensions, it is known that

$$\left\langle (x_t-x_0)^2\right\rangle = 4Dt. \quad (1)$$

The left-hand side of this equation is the mean squared displacement (MSD) in time t. Using data from the study described in Kluiters et al. [S6], we obtained an estimate for D of 0.531. This corresponds to an average distance of approximately 1.5 km per day.

$C(r,t)$ is interpreted as the number of infectious vectors at distance r from the source farm t days after the source farm became infectious. This is used to calculate the average number of new cases arising on the target farm as a result of vector dispersal from the source farm, as in the previous model. However, the formula for the rate at which cases arise at farm i as a result of infection at farm j (η_{ij}) now becomes

$$\eta_{ij}=d_{Si}C(r_{ij},t)\beta_{vh}a_i(T).$$

As before, d_{Si} is the degree of susceptibility of farm *i*, β_{vh} is the probability of transmission from vector to host given an effective contact and $a_i(T)$ is the biting rate at farm *i* when the temperature is *T*.

S1.5 Detection mechanism modified

Detection of cases determines when and where movement restrictions are imposed. It is, therefore, important to accurately capture this process. In the previous model, the parameters (λ_D and λ_{DS}) governing the probability of detection were estimated from a single report in the literature in combination with the fixed prevalence curve we were then using within the model. We hoped to update these estimates, but were unable to find good quality data. There are a few reports that quote the number of animals or proportion of a herd showing clinical signs (e.g. [S7]). However, these rarely include the total number of animals that were infectious. As a result, it is impossible to calculate the number of *infectious* animals showing clinical signs. Also, this approach neglects the fact that (with the exception of dairy farmers) most farmers check their stock relatively infrequently (could be as little as once a week in late summer) and from a distance, only investigating further if an animal is showing severe clinical signs. Add to this the fact the some of the signs of BTV infection are common to other diseases and it is easy to see that slight illness in one animal might be dismissed as nothing requiring immediate attention, unless of course BTV had already been confirmed on another farm. So, the probability of detection is really a combination of the proportion of animals showing severe clinical signs and the frequency with which animals are checked. To try and capture this we have introduced pre- and post-detection parameter values. Prior to the first case of BTV being identified, the pre-detection values are used. Afterwards, the model switches to the post-detection values. The actual values were chosen during the validation step (as described below).

S2. Validation

BTV model functions, parameters and default values are given in Table 3 in the main manuscript. Most were obtained from the literature (Gubbins et al. [S8] and references therein). Others were estimated during validation as described below. Additional parameter values considered during validation and sensitivity analysis are listed in Table S1.

Table S1: Additional BTV model parameter values considered during validation and sensitivity analysis.

**Description**	**Parameters**	**Default and additional values**
Vector to host ratio<br>$m(t,T) = \exp(b_0 + p_1\sin(\theta(t - \psi_1)) + p_2\sin(2\theta(t - \psi_2)) + cT)$,<br>where<br>$t$ = time<br>T = temperature	b_0, p_1, p_2, c, θ, ψ_1, ψ_2	**Set 1**: 0, 8.33, 3.04, 0.07, 0.0172, 128.4, 81.7<br>**Set 2 (default)**: 0, 10.59, 3.71, 0.07, 0.0172, 128.4, 81.7<br>**Set 3**: 0, 11.55, 3.98, 0.07, 0.0172, 128.4, 81.7<br>**Set 4**: 0, p_{14}, p_{24}, 0.07, 0.0172, 128.4, 81.7<br>where<br>$p_{14} \in \{6, 7, 8, 9, 10, 11, 12, 13, 14, 15\}$<br>$p_{24} = 0.35p_{14}$

Probability of detection<br>$1-(1-\lambda_A d_I)^H$,<br>where<br>$\lambda_A = \lambda_{D0}$ (or λ_{D1}) for cattle and mixed farms prior to (or after) detection of the first case.<br>Similarly for sheep with λ_{DS0} and λ_{DS1}.<br>d_I = prevalence of infection on the farm<br>H = herd size	λ_{D0}, λ_{DS0}, λ_{D1}, λ_{DS1}<br>(reflect proportion of infected animals that show severe clinical signs and frequency of checking by farmer)	**Set 1:** 0.001, 0.001, 0.001, 0.001<br>**Set 2 (default):** 0.001, 0.001, 0.01, 0.01<br>**Set 3:** 0.001, 0.001, 0.16, 0.9<br>**Set 4:** 0.01, 0.01, 0.01, 0.01<br>**Set 5:** λ_{D05}, λ_{DS05}, λ_{D15}, λ_{DS15}<br>where<br>$\lambda_{D05} \in \{0.0001, 0.001, 0.0025, 0.005, 0.0075, 0.01, 0.025, 0.05, 0.075, 0.1\}$<br>$\lambda_{DS05} = \lambda_{D05}$<br>$\lambda_{D15} \in \{0.0001, 0.001, 0.0025, 0.005, 0.0075, 0.01, 0.025, 0.05, 0.075, 0.1\}$<br>$\lambda_{DS15} = \lambda_{D15}$

S2.1 Within-farm model

To test the within-farm model, infection was initiated with either 1 latent or 1 infectious vector on day 217 (i.e. 5 August, the estimated date of introduction of the 2007 UK outbreak). Using the default parameter values, animal movement data from 2006 (i.e. the nearest disease-free year) and temperature data from 2007, the curves shown in Figure S3 were produced.

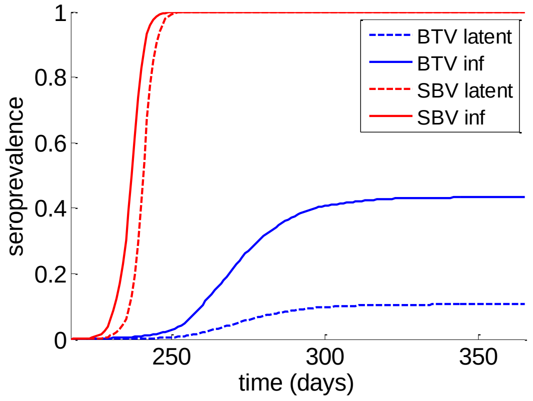

Figure S3: Seroprevalence curves for BTV (blue lines) and Schmallenberg virus (SBV, red lines) generated by the within-farm model when infection was introduced on 5 August 2007. Infection was initiated by introducing either one latent vector (dotted lines) or one infectious vector (solid lines).

Table S2: Within-herd seroprevalence estimates

**Estimate**	**Range**	**Species**	**Comment (Reference)**
8.9%	0.3 – 49.2	cattle	Interim measurement [S9]
3.6%	0.05 – 13.0	sheep	Interim measurement [S9]
11%	1 – 41	beef	Final measurement [S10]
11%	1 – 57	dairy	Final measurement [S10]
2%		mixed	Final measurement [S10]

Rather than prevalence, the curves show seroprevalence (i.e. the cumulative number of infected animals) as the tests used during the outbreak detect antibodies and not necessarily active infection. For BTV, the model gives a final seroprevalence of either 10.7% or 43.4%, depending on the nature of the introduction. As shown in Table S2, initial estimates during the UK outbreak were 8.9% (0.3 –

49.2) for cattle and 3.6% (0.05 – 13.0) for sheep [S9]. At the end of the outbreak and after further testing, the estimate for cattle was revised to 11% (1 – 57) [S10]. These are consistent with our estimates following the introduction of one latent vector.

In order to further validate the within-farm model, the exercise was repeated using parameter values for Schmallenberg virus (SBV), which is transmitted by the same vectors. See Table S3 for a comparison of BTV and SBV parameter values. The results for SBV show that the seroprevalence quickly tends to 100%, exceeding 90% at either 29 days post infection (dpi) or 25 dpi, depending on the mode of introduction. This is consistent with results published in Wernike et al. [S11], which show seroprevalence on a farm in Germany (close to the original source of the SBV outbreak) rising to 100% within 4 weeks of the first infected animal being detected.

Table S3: Comparison of BTV and SBV parameter values used in the within-farm model. Unless shown below, parameter values were as for BTV in Table S1.

Parameter	Symbol	BTV	SBV
host incubation rate (cattle)	c_C	1/7	1/2
host incubation rate (sheep)	c_S	1/5	1/2
recovery rate (cattle)	r_C	1/20.6	1/3.04
recovery rate (sheep)	r_S	1/16.4	1/4.37
probability of transmission from vector to host	b	0.9	0.76
probability of transmission from host to vector	β	0.01	0.14
rate at which exposed vectors become infectious	$v(T)$		
$v(T)=\max(0,0.019(T-13.34))$		Y	N
$v(T)=\max(0,0.03(T-12.35)$		N	Y

S2.2 Between-farm model

To test the main (i.e. between-farm) model, infection was introduced by making nine farms in Suffolk and Essex latently-infected on 5 August (Table S4). The date and places of introduction were based on information in Gloster et al. [S12] and the BTV case data for the 2007 UK outbreak. The index cases were farms that were found to be infected within one week of the first reported case. For reasons given below, we assumed that they were all exposed on the same night.

Table S4: Details of BTV cases reported in the first week of the 2007 UK outbreak. Precise locations were used to initiate infection in the BTV model during validation.

County	Date reported
Suffolk	21/09/07
Suffolk	23/09/07
Suffolk	24/09/07
Suffolk	28/09/07
Suffolk	28/09/07
Suffolk	28/09/07
Essex	28/09/07
Essex	27/09/07
Suffolk	28/09/07

Reasons:

- If we assume infectious midges were blown over from mainland Europe, then we started with one (or more) exposed animals on one or more farms.
- The average time it takes an exposed animal to become infectious ('time to first infectious farm') equals the average host incubation period (HIP), which is 7 days for cattle and 5 days for sheep.
- At best, detection is likely to occur several days later.
- The average time from infectious animals appearing on the primary farm to infectious animals appearing on another (secondary) farm as a result of infection on the primary farm (i.e. 'time to secondary infectious farm') must equal the sum of the average extrinsic incubation period (EIP) and the average HIP.
- EIP varies with temperature, but in September in the UK is likely to be 6 – 14 days.
- So, the average 'time to secondary infectious farm' equals 11 – 21 days.
- Therefore, it is likely that all farms found to be infected within one week of the first reported case were exposed on the same night as the first case, as insufficient time had elapsed for them to be secondary cases.

As discussed above, the detection parameters could not be estimated from data and so were selected as follows:

- The pre-detection values had to be low enough to give a median date of first detection that was close to the observed date of 21 September (following introduction on 5 August).
- The post-detection values had to be such that the median number of farms detected by the end of the year was approximately 42 (the number reporting clinical signs during the UK outbreak).

Note that the total number of farms infected during the outbreak (i.e. all farms identified on or before 15 March 2008) was 135. Around 25 of these were detected by a combination of surveillance and tracing. It is impossible to say how many might have been detected later as a result of clinical signs. However, the fact that approximately half of all cases were only found following pre-movement testing in 2008 suggests that many of them would have gone undetected.

Unfortunately, 8 cases of foot-and-mouth disease (FMD) were also reported in the UK in 2007. For the purposes of validation only, FMD movement restrictions were approximated within the model by removing all animal movements for two periods:
1) 3 August to 7 September (day 215 to 250)
2) 12 September to 4 October (day 255 to 277).

Through a process of trial and error, rather than fitting, we found that by using 0.001 before the first case was detected (i.e. $\lambda_{D0} = \lambda_{DS0} = 0.001$) and 0.01 thereafter (i.e. $\lambda_{D1} = \lambda_{DS1} = 0.01$) we obtained results consistent with the observed data. Starting with nine latently-infected index cases on the 5 August (as described above), the median total number of farms infected was 118 (min 95 - max 261) with 37 (min 27 - max 52) detected by the end of the year (see Table S5). The first case was detected on 10 Sep (min 12 Aug - max 28 Sep). The number of farms detected each week is shown in Figure S4. Increasing or decreasing the post-detection values (λ_{D1}, λ_{DS1}) affects the total number of farms detected, but has little effect on the total number of farms infected. Naturally, it has no effect on the date of first detection. Table S5 also contains results for the case where the pre-detection values (λ_{D0}, λ_{DS0}) are both 0.01 (i.e. equal to the default post-detection values). While the numbers of farms infected and detected were unchanged, the date of first detection was far too early (the maximum was almost two weeks earlier than the actual date).

Table S5: Median values (min and max shown in brackets) for the total numbers of farms infected and detected and date of first detection. Results generated with and without FMD movement restrictions.

		BTV restrictions only			BTV and FMD restrictions		
**Pre-detection λ_{D0}, λ_{DS0}**	**Post-detection λ_{D1}, λ_{DS1}**	**total number of farms infected**	**total number of farms detected**	**date of first detection**	**total number of farms infected**	**total number of farms detected**	**date of first detection**
0.001, 0.001	0.001, 0.001	120 (98-244)	9 (4-17)	10 Sep (12 Aug – 7 Oct)	118 (96-337)	8 (3-16)	10 Sep (12 Aug – 28 Sep)
0.001, 0.001	0.01, 0.01	119 (95-252)	37 (26-71)	10 Sep (12 Aug – 7 Oct)	118 (95-261)	37 (27-52)	10 Sep (12 Aug – 28 Sep)
0.001, 0.001	0.16, 0.9	118 (102-258)	114 (98-249)	10 Sep (12 Aug – 7 Oct)	116 (102-138)	113 (97-135)	10 Sep (12 Aug – 28 Sep)
0.01, 0.01	0.01, 0.01	121 (100-219)	38 (27-62)	23 Aug (12 Aug – 9 Sep)	118 (97-140)	37.5 (27-52)	24 Aug (12 Aug – 9 Sep)

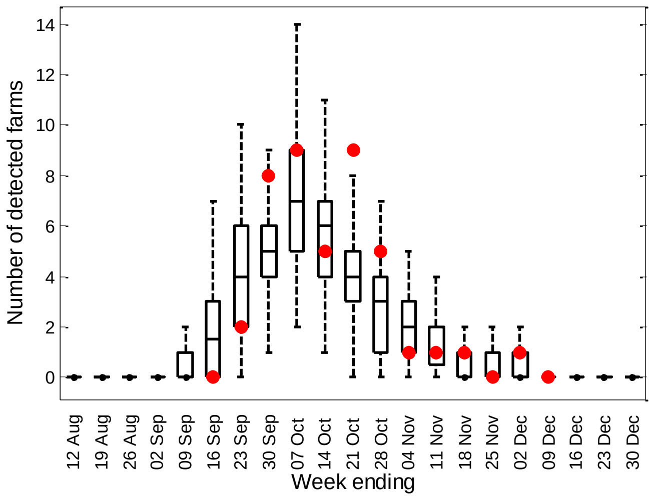

Figure S4: Number of farms detected per week. Boxplots are based on 100 simulations of the model. The red points indicate the numbers of farms confirmed as positive after reporting clinical signs.

In terms of spatial distribution, Figure S5 shows each farm's probability of detection (i.e. the proportion of simulations in which they were detected). Individual simulations in which the number of farms detected equals the median and maximum values of 500 simulations, respectively, are also shown along with a plot of cases reported in 2007.

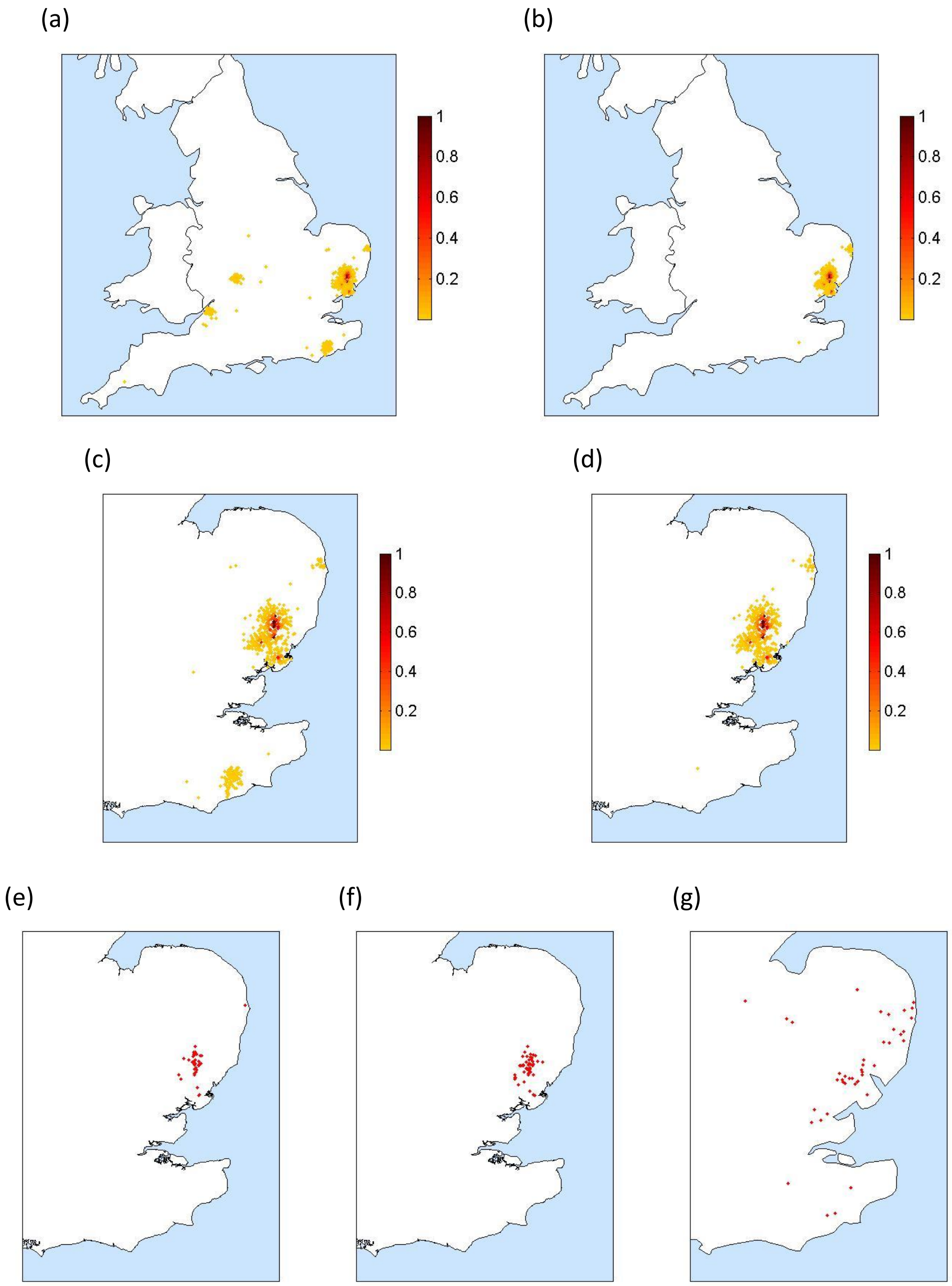

Figure S5: (a) – (d) Maps of England and Wales and southeast England showing the probability of detection (based on 500 simulations) when infection is introduced into Suffolk and Essex on 5 August: (a) & (c) with BTV movement restrictions only; (b) & (d) with BTV and FMD movement restrictions. (e) & (f) Spatial distribution of farms detected in the 'median' and 'maximum' simulations generated using BTV and FMD movement restrictions. (g) Spatial distribution of farms confirmed after reporting clinical signs in 2007. Maps were produced using Matlab version 7.12.0.635 (R2011a, www.mathworks.com).

The model predictions show a more clustered pattern of cases than that observed. This could be due to some movements being omitted (e.g. illicit movements, which are not recorded in the movement databases) or the way in which we have modelled the diffusion of vectors (e.g. without including the effects of wind). It could be because we do not allow movements from high risk to low risk zones. In

reality, these are allowed if the source farm tests negative. In terms of pathogen transmission, this means that we could be preventing the movement of latently-infected animals, which would otherwise be allowed. This is only an issue after the first case has been detected. Prior to that, long-range movement of infection can occur as shown in Figures S5a and S5b. There is a small chance that the lack of dispersal could be due to the FMD restrictions in our model. These are approximate. Figure S6 shows that small numbers of movements occurred within the periods during which we prevented all movements, in particular between 24 Aug (when movements outside the FMD Surveillance Zone were allowed subject to additional controls) and 7 Sep (after which all FMD restrictions were lifted). These movements occurred before BTV was detected and BTV movement restrictions were implemented. So, there is a chance that they could have resulted in dissemination of BTV that is not captured by our model. However, the results of tracing and testing carried out by Defra [S10] suggest that this is not very likely. Finally, the relatively wide scattering of observed cases could be due to additional (i.e. on the same night) or subsequent introductions. Gloster et al. [S12] shows that several other nights in August, September and October were considered suitable for vector incursion and it has been stated that it is likely that several incursions took place between 4th August and 20th December 2007 [S10].

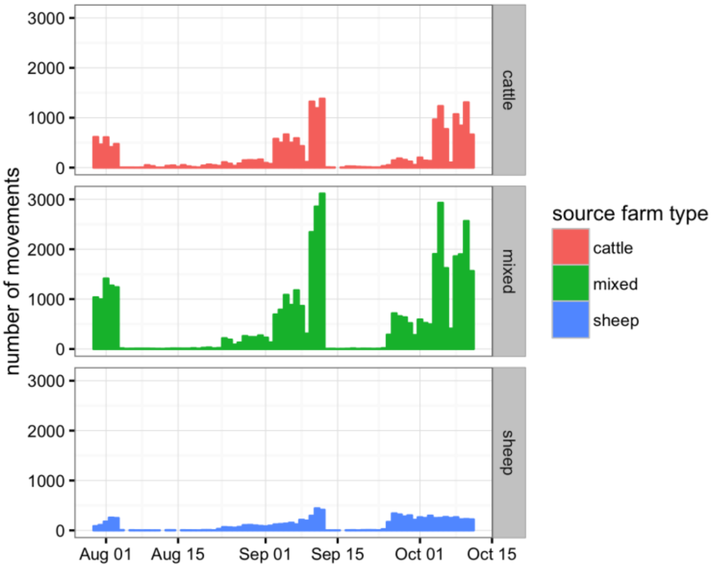

Figure S6: Number of animal movements per day in England and Wales in 2007 broken down by source farm type.

S2.3 Additional simulations of between-farm model

In order to verify that no other combinations of λ_{D0}, λ_{DS0}, λ_{D1} and λ_{DS1} were capable of producing results consistent with the outbreak data, particularly when p_1 and p_2 were also allowed to vary, an additional 100,000 simulations were executed. Parameter values for p_1 and p_2 (Set 4 in Table S1) were combined with values for λ_{D0}, λ_{DS0}, λ_{D1} and λ_{DS1} (Set 5 in Table S1) to produce 1000 parameter sets. For each parameter set, 100 simulations were run and three median values were calculated, namely the median number of farms infected, the median number of farms detected and the median date of first detection. For each of these, a feasible range (criterion) based on the observed value was specified. Only parameter sets that satisfied all three criteria were retained. The criteria are listed below and the results presented in Table S6 and Figure S7.

Criteria:
Median number of farms infected must be in the interval [80, 150] (observed = 135).
Median number of farms detected must be in the interval [20, 60] (observed = 42).
Median day of first detection must be in the interval [7 Sep, 7 Oct] (observed = 21 Sep).

Table S6: Parameter sets that produced results which satisfied all three criteria.

p_1	λ_{D0}	λ_{D1}	**Median number infected**	**Median number detected**	**Median day of first detection**
10	0.001	0.0075	84	20	256
10	0.001	0.01	83	23	256
10	0.001	0.025	82	39	256
10	0.001	0.05	83	53	256
11	0.001	0.0025	148	21	252
11	0.001	0.005	148.5	34	252
11	0.001	0.0075	148.5	41	252
11	0.001	0.01	149	51	252

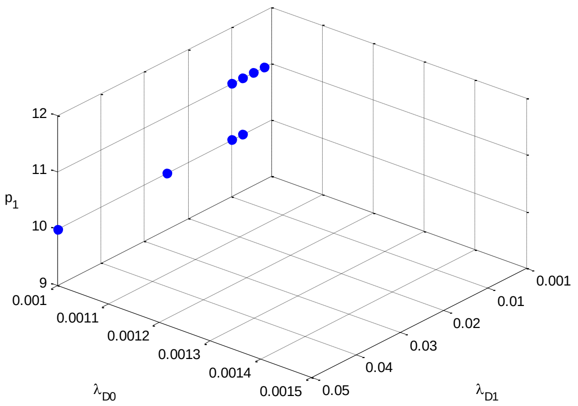

Figure S7: Each point corresponds to a parameter set that produced results which satisfied all three criteria.

It is clear from Table S6 and Figure S7 that the parameter values selected in the previous subsection and used throughout this paper as the default values, namely p_1 = 10.59, λ_{D0} = 0.001 and λ_{D1} = 0.01, are consistent with these results. Furthermore, only combinations involving closely related values can produce results consistent with the outbreak data.

S3. Sensitivity analysis

S3.1 Effect of different *ma* values

As explained in section 1.3, the product of the new seasonal vector-to-host ratio (*m*) and the temperature-dependent biting rate (*a*) reasonably represents the pattern of trap catch data, in particular the summer plateau. For validation purposes, we used parameter values that produce a plateau approximately equal to 500 biting midges per host per day, which could be considered a conservative estimate. As the model is sensitive to *m*, and hence *ma*, here we present results for scenarios in which the plateau is approximately equal to 100 and 1000 too. Figure S8 shows plots of *ma* versus time. The blue and green curves were generated using daily temperature data (with 5-day smoothing) from 2006 and 2007, respectively. The red curve was produced using data corresponding to the mean across years 1995-2014. As indicated by the horizontal black lines, the plateau height of

the mean curve is approximately 100, 500 and 1000, respectively. Essentially, the figures are identical except for the height of the plateaux.

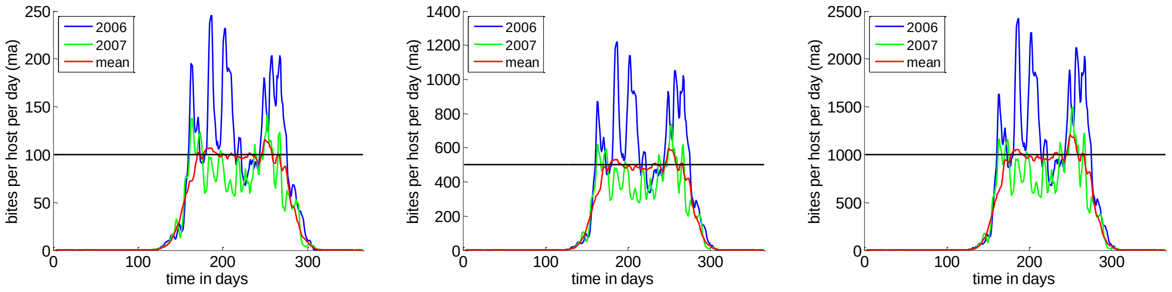

Figure S8: Plots of *ma* vs time with the plateau of the mean curve approximately equal to 100, 500 and 1000, respectively.

To facilitate direct comparison with the validation results, the first set of sensitivity results (Tables S7 and S8) was generated using the default parameter values (except for the two controlling the plateau height and the four detection parameters), nine specified index cases on 5 August in Suffolk and Essex, 2006 movement data and 2007 temperature data. The second set of sensitivity results (Tables S9 and S10) was produced using the same parameter values, one random index case on 1 June in Hampshire, 2013 movement data and 'average' temperature data. The 'average' temperature data corresponds to data from one year (2011) with a mean across the summer months (MJJASO) that closely matches the median of summer values over the last 10 years (1996 – 2015). These results are useful as they reveal the behaviour of the model when using a different starting time, a different starting place, different movement and temperature data and a different number of index cases. These settings are also used to produce some of the results in the main paper.

Table S7: First set of sensitivity results. Median values (min – max) for the total numbers of farms infected when the plateau of the *ma* curve approximately equals 100, 500 and 1000, respectively. Results were generated with BTV movement restrictions only.

		***ma* = 100**	***ma* = 500**	***ma* = 1000**
**Pre-detection λ_{D0}, λ_{DS0}**	**Post-detection λ_{D1}, λ_{DS1}**	**total number of farms infected**	**total number of farms infected**	**total number of farms infected**
0.001, 0.001	0.001, 0.001	23 (14-43)	120 (98-244)	216.5 (176-442)
0.001, 0.001	0.01, 0.01	23 (14-43)	119 (95-252)	215 (180-429)
0.001, 0.001	0.16, 0.9	23 (14-43)	118 (102-258)	212 (167-427)
0.01, 0.01	0.01, 0.01	22 (14-41)	121 (100-219)	215.5 (174-452)

Table S8: First set of sensitivity results. Median values (min – max) for the total numbers of farms detected and date of first detection when the plateau of the *ma* curve approximately equals 100, 500 and 1000, respectively. Results were generated with BTV movement restrictions only.

		***ma* = 100**		***ma* = 500**		***ma* = 1000**	
**Pre-detection λ_D, λ_{DS}**	**Post-detection λ_D, λ_{DS}**	**total number of farms detected**	**date of first detection**	**total number of farms detected**	**date of first detection**	**total number of farms detected**	**date of first detection**
0.001, 0.001	0.001, 0.001	1 (0-4)	27 Sep (12 Aug – 12 Dec)	9 (4-17)	10 Sep (12 Aug – 7 Oct)	19 (12-33)	5 Sep (12 Aug – 17 Sep)
0.001, 0.001	0.01, 0.01	4 (0-10)	27 Sep (12 Aug – 12 Dec)	37 (26-71)	10 Sep (12 Aug – 7 Oct)	80 (59-134)	5 Sep (12 Aug – 17 Sep)
0.001, 0.001	0.16, 0.9	16.5 (0-28)	27 Sep (12 Aug – 12 Dec)	114 (98-249)	10 Sep (12 Aug – 7 Oct)	206 (164-417)	5 Sep (12 Aug – 17 Sep)
0.01, 0.01	0.01, 0.01	6 (2-15)	22 Aug (12 Aug – 29 Sep)	38 (27-62)	23 Aug (12 Aug – 9 Sep)	80.5 (59-137)	22 Aug (12 Aug – 2 Sep)

Table S9: Second set of sensitivity results. Median values (min – max) for the total numbers of farms infected when the plateau of the *ma* curve approximately equals 100, 500 and 1000, respectively. Results were generated with BTV movement restrictions only.

		***ma* = 100**	***ma* = 500**	***ma* = 1000**
**Pre-detection λ_{D0}, λ_{DS0}**	**Post-detection λ_{D1}, λ_{DS1}**	**total number of farms infected**	**total number of farms infected**	**total number of farms infected**
0.001, 0.001	0.001, 0.001	10 (1-249)	414 (1-2327)	798 (1-6339)
0.001, 0.001	0.01, 0.01	10 (1-254)	418.5 (1-1948)	837.5 (1-5878)
0.001, 0.001	0.16, 0.9	10 (1-239)	412 (1-1991)	820 (1-5103)
0.01, 0.01	0.01, 0.01	12 (1-254)	361.5 (1-1092)	700.5 (1-2036)

Table S10: Second set of sensitivity results. Median values (min – max) for the total numbers of farms detected and date of first detection when the plateau of the *ma* curve approximately equals 100, 500 and 1000, respectively. Results were generated with BTV movement restrictions only.

		ma = 100		*ma* = 500		*ma* = 1000	
**Pre-detection λ_D, λ_{DS}**	**Post-detection λ_D, λ_{DS}**	**total number of farms detected**	**date of first detection**	**total number of farms detected**	**date of first detection**	**total number of farms detected**	**date of first detection**
0.001, 0.001	0.001, 0.001	0 (0-14)	26 Sep (13 Jun – 8 Dec)	48.5 (0-171)	11Aug (13 Jun – 2 Nov)	131 (0-951)	3 Aug (13 Jun – 17 Sep)
0.001, 0.001	0.01, 0.01	0 (0-77)	26 Sep (13 Jun – 8 Dec)	171.5 (0-607)	11Aug (13 Jun – 2 Nov)	402.5 (0-2756)	3 Aug (13 Jun – 17 Sep)
0.001, 0.001	0.16, 0.9	0 (0-234)	26 Sep (13 Jun – 8 Dec)	397 (0-1929)	11Aug (13 Jun – 2 Nov)	793 (0-4966)	3 Aug (13 Jun – 17 Sep)
0.01, 0.01	0.01, 0.01	2 (0-77)	13 Aug (9 Jun – 18 Nov)	151.5 (0-549)	19 Jul (9 Jun – 27 Aug)	342.5 (0-992)	15 Jul (9 Jun – 16 Aug)

In Table S9, when the pre- and post-detection parameters are (0.001, 0.001) and (0.01, 0.01) respectively, the median number of farms infected is 10 when the plateau of *ma* is approximately equal to 100, and 837.5 when the plateau is approximately equal to 1000. This illustrates the sensitivity of the model to the number of bites per host per day.

S3.2 Effect of detection parameters

Detection rates were revisited as part of the validation step, where results for a range of values were presented. Detection rates affect the timing of control measures, yet there is little information about them. Additional results with different levels of *ma* and different initial and running conditions are included in Tables S7 – S10.

S3.3 Partial Rank Correlation Coefficients

To assess the sensitivity of the model to small perturbations in parameter values, we calculated the partial rank correlation coefficient (PRCC) of each parameter with respect to the median outbreak size. This method allows parameter values to be varied simultaneously. As we did not have feasible distributions for each parameter, we chose to use Uniform distributions with limits equal to +/- 10% of the default estimate in each case. 100 parameter sets were created using Latin Hypercube Sampling. For each parameter set, the model was run 100 times. Each time, infection was introduced into Hampshire on 1 June and 2013 movement data and average temperature data were used. The results are presented in Table S11 with parameters considered to be significant shown in bold. Top of the list is p_1, which is the main parameter governing the height of the *ma* plateau. This is followed by v_2, the virus' development threshold, and μ_2, one of the parameters controlling vector mortality. The remaining parameters in bold include those that determine the vector biting rate, the effect of temperature on *m*, the host to vector transmission probability and vector diffusion. This clearly demonstrates that median outbreak size is largely determined by the level of vector transmission.

Table S11: Partial rank correlation coefficients (PRCC) and associated p-values for median outbreak size with each model parameter. Parameters considered to be significant are shown in bold.

Parameter	Median outbreak size	p-value
p_1	**0.9324**	**0**
v_2	**-0.9112**	**0**
μ_2	**-0.6330**	**5e-09**
a_2	**-0.6006**	**5e-08**
a_1	**0.5083**	**8e-06**
μ_1	**-0.4705**	**5e-05**
c	**0.4067**	**0.0005**
a_4	**0.3949**	**0.0008**
β_{hv}	**0.3827**	**0.0012**
D	**0.3095**	**0.0096**
β_{vh}	0.3030	0.0114
ψ_1	-0.2978	0.0129
λ_{DS0}	-0.2682	0.0259
vecd1	0.2345	0.0524
a_3	-0.2311	0.0561
r_S	-0.1632	0.1804
σ	0.1417	0.2453
prevE	-0.1206	0.3235
r_C	-0.1052	0.3895
k_C	0.0789	0.5192
λ_{DS1}	-0.0717	0.5585
λ_{DC1}	-0.0617	0.6146
ψ_2	0.0578	0.6368
p_2	-0.0562	0.6462
v_1	0.0539	0.6599
λ_C	0.0493	0.6872
mixed_ratio	-0.0479	0.6957
c_S	-0.0453	0.7116
λ_I	-0.0371	0.7623
λ_{DC0}	0.0188	0.8781
c_C	0.0173	0.8880
k_I	-0.0155	0.8995

S4. Additional results

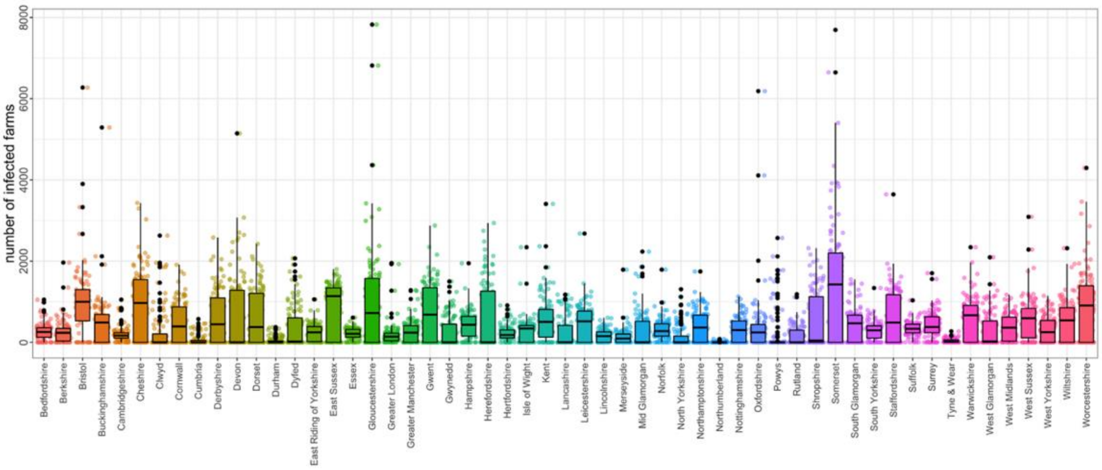

Figure S9: Box plots showing the number of farms infected in England and Wales by county of introduction (i.e. OS ceremonial county in which the index case was located). Results are based on 100 simulations per county.

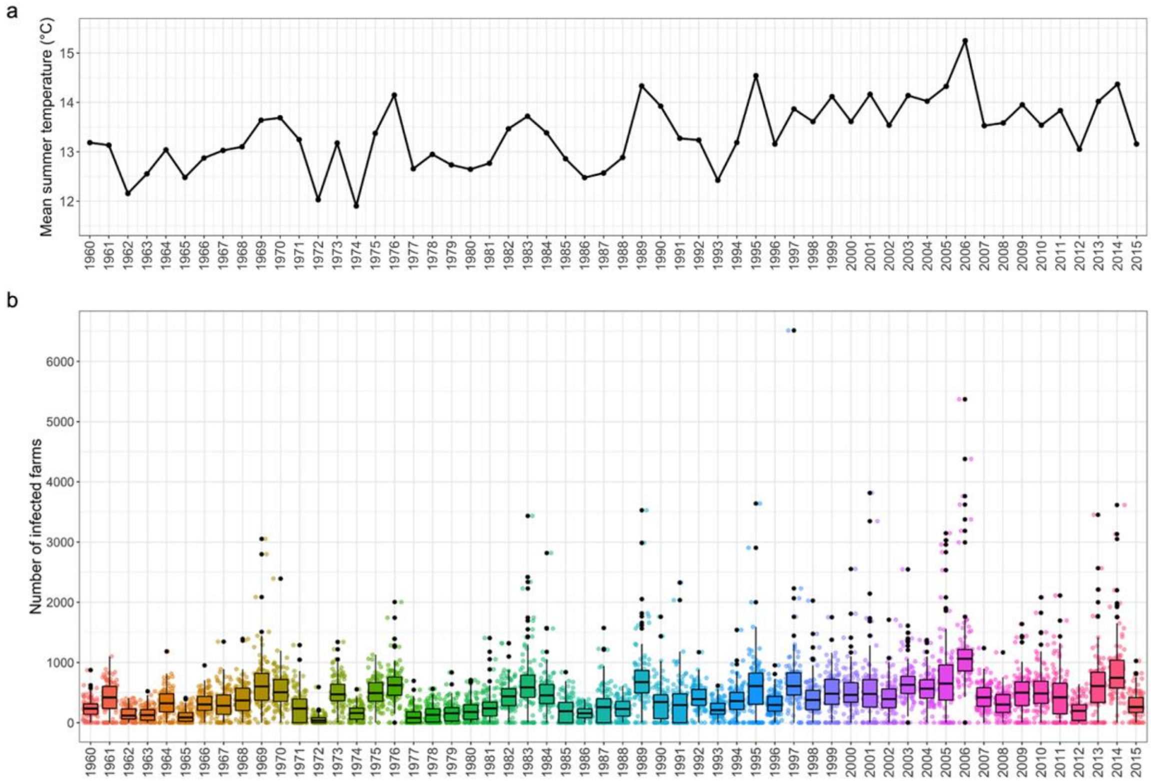

Figure S10: (a) Mean summer temperature (°C) in England and Wales from 1960 to 2015. Daily temperature data covering May to October for each grid point containing at least one farm with cattle and sheep were used to calculate the mean in each case. (b) Model output showing the total number of farms infected when using the corresponding temperature data.

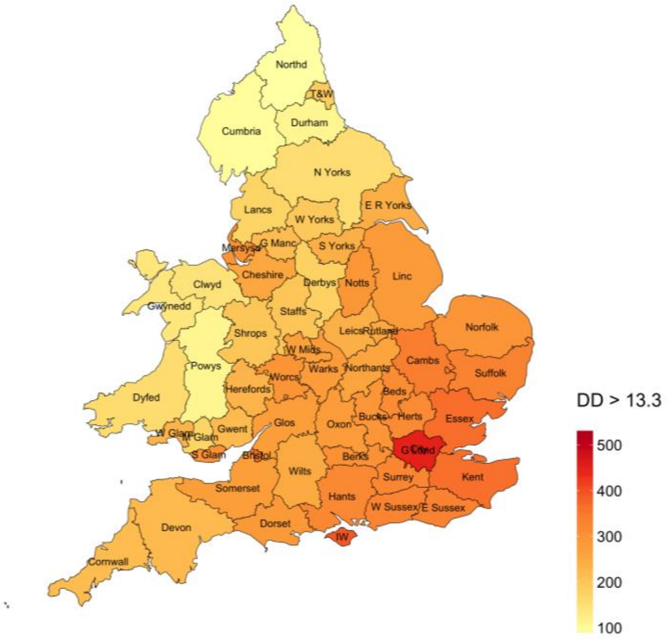

Figure S11: County level values for the number of DD > 13.3°C in 2007.

References

S1. Turner, J., Bowers, R. G. & Baylis, M. Modelling bluetongue virus transmission between farms using animal and vector movements. *Sci. Rep.* **2**, 319; DOI:10.1038/srep00319 (2012).

S2. Jenkins, G. J., Perry, M. C. & Prior, M. J. The climate of the United Kingdom and recent trends. Met Office Hadley Centre, Exeter, UK (2008).

S3. Sanders, C. J. et al. Influence of season and meteorological parameters on flight activity of *Culicoides* biting midges. J. Appl. Ecol. **48**, 1355-1364; DOI:10.1111/j.1365-2664.2011.02051.x (2011).

S4. Meiswinkel, R. et al. The Mondrian matrix: *Culicoides* biting midge abundance and seasonal incidence during the 2006–2008 epidemic of bluetongue in the Netherlands. *Med. Vet. Entomol.* **28**, 10-20; DOI:10.1111/mve.12013 (2014).

S5. Crank, J. The Mathematics of Diffusion (2nd Edition). Clarendon Press, Oxford (1975).

S6. Kluiters, G., Swales, H. & Baylis, M. Local dispersal of palaearctic *Culicoides* biting midges estimated by mark-release-recapture. *Parasites & Vectors* **8**, 86; DOI:10.1186/s13071-015-0658-z (2015).

S7. Elbers, A. R. W. et al. Field observations during the Bluetongue serotype 8 epidemic in 2006 II. Morbidity and mortality rate, case fatality and clinical recovery in sheep and cattle in the Netherlands. *Prev. Vet. Med.* **87**, 31-40; DOI:10.1016/j.prevetmed.2008.06.003 (2008).

S8. Gubbins, S. et al. Inferences about the transmission of Schmallenberg virus within and between farms. *Prev. Vet. Med.* **116**, 380–390; DOI:10.1016/j.prevetmed.2014.04.011 (2014).

S9. Defra. Initial epidemiological report on the outbreak of bluetongue in East Anglia and South East England from investigations completed to 19 October 2007. Defra, UK (2007).

S10. Defra. Report on the distribution of bluetongue infection in Great Britain 15 March 2008. Defra, UK (2008).

S11. Wernike, K., Silaghi, C., Nieder, M., Pfeffer, M., Beer, M. Dynamics of Schmallenberg virus infection within a cattle herd in Germany, 2011. *Epidemiol. Infect.* **142**, 1501-1504; DOI:10.1017/S0950268813002525 (2014).

S12. Gloster, J., Burgin, L., Witham, C., Athanassiadou, M. & Mellor, P. S. Bluetongue in the United Kingdom and northern Europe in 2007 and key issues for 2008. *Vet. Rec.* **162**, 298-302 (2008).

Acknowledgements

This work was funded by BBSRC grant BB/J015806 awarded to MB and APM. The authors would like to thank Ian Smith of the Computing Services Department, University of Liverpool for performing some of the necessary computations on the Amazon Web Services (AWS). We acknowledge support from the AWS Cloud Credit for Research given to the University of Liverpool as part of a collaboration with Dell Corporation Limited, Alces Software Limited and the University. The UK Climate Projections (UKCP09) have been made available by the Department for Environment, Food and Rural Affairs (Defra) and the Department of Energy and Climate Change (DECC) under licence from the Met Office, UKCIP, British Atmospheric Data Centre, Newcastle University, University of East Anglia, Environment Agency, Tyndall Centre and Proudman Oceanographic Laboratory. These organisations give no warranties, express or implied, as to the accuracy of the UKCP09 and do not accept any liability for loss or damage, which may arise from reliance upon the UKCP09 and any use of the UKCP09 is undertaken entirely at the users risk. The authors would like to thank Debbie Hemming and Mike Kendon at the Met Office for providing the UKCP09 data. They would also like to thank APHA for providing the agricultural census, animal movement and BTV case data. UK county boundaries were downloaded from OS OpenData (https://www.ordnancesurvey.co.uk). Contains OS data © Crown copyright and database right (2017).
